# Acute and long-term effects of repetitive transcranial magnetic stimulation in major depressive episodes: a systematic review and dose-response meta-analysis of randomized sham-controlled trials

**DOI:** 10.1186/s12916-026-05089-z

**Published:** 2026-07-30

**Authors:** Zuxing Wang, Ruanmei Sheng, Ruifeng Shi, Zhili Zou, Vaughn R. Steele, Tifei Yuan, Xiaoyun Guo

**Affiliations:** 1 Shanghai Key Laboratory of Mental Disorders Translational Research, Brain Health Institute, Shanghai Mental Health Center, School of Psychology, National Center for Mental Disorders, Shanghai Jiao Tong University School of Medicine and School of Psychology, Shanghai, 201108 China; 2 Sichuan Provincial People’s Hospital, Sichuan Provincial Center for Mental Health, University of Electronic Science and Technology of China, Chengdu, 611130 China; 3 Songjiang Hospital Affiliated to Shanghai Jiao Tong University School of Medicine, Shanghai, 201600 China; 4 Zhejiang Key Laboratory of Precision Psychiatry, Hangzhou, 310003 China; 5 School of Medicine, Department of Psychiatry, Yale University, New Haven, CT 06510 USA; 6 Olin Neuropsychiatry Research Center, Institute of Living, Hartford Hospital/HealthCare, Hartford, CT 06114 USA; 7 Center for Brain & Mind Health, Yale University, New Haven, CT 06519 USA; 8 Co-innovation Center of Neuroregeneration, Nantong University, Nantong, Jiangsu 226019 China

**Keywords:** Transcranial magnetic stimulation, Major depressive episodes, Acute efficacy, Follow-up, Dose-response relationship

## Abstract

**Background:**

To characterize the dose-response relationships between key repetitive transcranial magnetic stimulation (rTMS) dosing parameters and clinical outcomes in major depressive episodes (MDE), and to identify group-level dose ranges at which predicted benefits plateaued for each dosing dimension separately.

**Methods:**

We performed a systematic review and dose-response meta-analysis of randomized sham-controlled trials investigating rTMS for adults with MDE. Outcomes were symptom severity, response, and remission, assessed acutely and in follow-up (> 7 days). Four key dosing dimensions (total pulses, pulses/session, sessions, duration) were examined separately using restricted cubic spline models, and the dose associated with the highest predicted group-level benefit within the observed range was derived from the fitted model. Effect sizes were expressed as standardized mean differences and risk ratios with 95% confidence intervals.

**Results:**

Across 108 trials (134 active arms; *n* = 5,621), total pulses, pulses per session, total sessions and treatment duration showed significant non-linear associations with acute efficacy in separate univariable models. For each dosing dimension considered separately, the highest predicted group-level acute benefits were observed at approximately 30,000–39,000 total pulses, 1,800–2,200 pulses per session, 14–16 sessions, and 2.7–3.1 weeks of treatment, with limited additional benefit from higher exposures. Follow-up analyses showed smaller and mostly linear associations, with the most favorable group-level estimates generally observed at 26,000–33,000 total pulses, 1,300–1,800 pulses per session, 10–14 sessions, and 2.8–3.3 weeks of treatment. Sensitivity analyses confirmed the stability of these estimates, and publication bias was minimal.

**Conclusions:**

In separate univariable models, rTMS efficacy in MDE appeared to plateau within moderate dose ranges for each dosing dimension. These findings provide descriptive group-level reference points rather than an individualized dosing rule or a single optimal treatment protocol. Sustained benefit may depend more on maintenance strategies than on further dose escalation alone.

**Supplementary Information:**

The online version contains supplementary material available at https://doi.org/10.1186/s12916-026-05089-z.

## Background

Major depressive episode (MDE) remains a leading cause of disability worldwide and imposes substantial clinical and economic burden [1]. Transcranial magnetic stimulation (TMS) is an established, guideline-endorsed, noninvasive treatment for depression [2]. However, clinical protocols vary widely in stimulation targets and dosing parameters (total pulses, pulses per session, number of sessions, and overall treatment duration), resulting in considerable heterogeneity in reported efficacy [3–6].

Recent dose-response syntheses point in complementary directions while leaving practical gaps [7–9]. In treatment-resistant depression (TRD), a multivariable meta-analysis reported that intensity, frequency, pulses per session, treatment duration, number of sessions, and total pulses explained meaningful variance [7]. Similarly, in TRD, a total-pulse-based analysis suggested a non-linear relationship peaking near 26,660 pulses, with frequency and age as significant moderators [8]. A trajectory-focused analysis showed a logarithmic improvement pattern with a plateau around weeks 3–4 and larger cumulative benefit at ≥ 3000 pulses per session and higher total pulses [9]. Additionally, a cross-protocol meta-analysis estimated near-maximal effective doses and identified bell-shaped or ascending dose-response curves across paradigms using end-of-treatment assessments, across multiple psychiatric disorders [10]. However, these studies differed in both scope and analytic strategy. Most focused on a single exposure metric, a limited set of outcomes, or end-of-treatment symptom assessments only, rather than examining multiple dose dimensions across both acute and follow-up outcomes while also incorporating binary clinical endpoints such as response and remission within a unified dose-response framework. To address these gaps, we conducted a dose-response meta-analysis of major depressive episodes to examine the nonlinear associations of total pulses, pulses per session, number of sessions, and treatment duration with acute and follow-up outcomes, including depressive-symptom severity, treatment response, and remission, with each dosing dimension analyzed separately to characterize group-level dose-response patterns rather than to derive a single optimal combined protocol.

## Methods

This systematic review and meta-analysis followed the Preferred Reporting Items for Systematic Reviews and Meta-Analyses (PRISMA) statement [11], and was prospectively registered in PROSPERO (registration number CRD420251051992).

### Search strategy

We systematically searched CENTRAL (Cochrane Central Register of Controlled Trials), Embase, and PubMed from inception to May 1, 2025, for randomized controlled trials (RCTs) of TMS in MDE, restricted to English-language publications. Reference lists of relevant reviews and articles were screened, and corresponding authors were contacted for missing data. Two reviewers independently screened titles, abstracts, and full texts in duplicate, resolving disagreements by discussion or a third reviewer. Complete search strategies are provided in the Additional File 1.

### Eligibility criteria

#### Participants (P)

Adults aged 18–65 years with a MDE in major depressive disorder (MDD) or bipolar depression, diagnosed by Diagnostic and Statistical Manual of Mental Disorders (DSM) [12] or International Classification of Diseases (ICD) [13]. Exclusion criteria included age < 18 or >65, secondary mood disorders, severe cognitive impairment, or relapse-prevention/maintenance designs.

#### Interventions (I)

Therapeutic TMS at any cortical site, frequency, or coil type. Eligible modalities included repetitive TMS (high-frequency, low-frequency, bilateral, accelerated), theta-burst stimulation (TBS: intermittent [iTBS] and continuous [cTBS]), and deep TMS. Trials in which TMS was not the primary intervention were excluded.

#### Comparators (C)

Sham TMS using accepted inert methods (e.g., sham coil or angled coil). For cross-over trials, only pre-cross-over data were used.

#### Outcomes (O)

The six prespecified primary outcomes were assessed at two time points: an acute phase (end of treatment or within 7 days after treatment completion) and a follow-up phase (more than 7 days after treatment completion). When multiple follow-up timepoints were reported, the assessment closest to 2 weeks post-treatment was prioritized for extraction. Each window included three endpoints: continuous depressive-symptom severity, treatment response, and remission. Symptom severity was preferentially extracted from Hamilton Rating Scale for Depression (HAMD) [14], Montgomery-Asberg Depression Rating Scale (MADRS) [15], or Beck Depression Inventory (BDI) [16]. Symptom severity was extracted according to a prespecified hierarchy: HAMD first, then MADRS, and BDI if neither clinician-rated scale was available. Binary endpoints (response and remission) were extracted as reported in each trial, adhering to the trial’s prespecified definitions and analyses. We did not derive response or remission from continuous data when not reported. Where provided, common criteria included ≥50% reduction from baseline for response and instrument-specific remission cut-offs (e.g., HAMD-17 ≤ 7, MADRS ≤ 10).

#### Study design (S)

Peer-reviewed RCTs comparing active versus sham TMS, with extractable data.

### Data extraction

Two reviewers independently extracted trial-level data using a piloted form, with disagreements resolved by a third reviewer. For each study arm, we recorded author, year, and country; intervention details (active TMS parameters and sham control method); diagnosis (major depressive episode in MDD or bipolar depression) and diagnostic criteria (DSM or ICD); treatment-resistance status; sample size (randomized and completed); sex distribution (male/female); age (mean, standard deviation [SD]); rating scale used; depression severity (mean, SD) at baseline, post-treatment, and follow-up; concomitant medication status and any washout period; stimulated brain region (no restriction; H1 deep TMS was operationally categorized as bilateral prefrontal stimulation with left-sided predominance, while acknowledging possible medial prefrontal engagement); stimulation frequency (Hz); intensity as % of motor threshold (MT%); pulses per session and number of sessions; total pulses (calculated when not explicitly reported as pulses-per-session × sessions); and treatment duration, defined as the total duration of the treatment course in weeks from the first to the last day of stimulation.

For studies with flexible treatment duration, treatment duration was characterized using the randomized acute treatment phase. When the actual treatment duration achieved during that phase was reported, we extracted the reported value; otherwise, we used the protocol-defined acute treatment duration for coding. Acute outcomes were extracted at the end of the randomized acute treatment phase, rather than from subsequent open-label or extension phases. Outcomes were extracted at post-treatment and follow-up. Follow-up was defined as any assessment conducted more than 7 days after treatment completion. When multiple follow-up assessments were reported, we prioritized the timepoint closest to 2 weeks post-treatment. Endpoints comprised continuous depressive-symptom severity and the binary outcomes of response and remission. Where necessary, missing dispersion metrics (e.g., SD) were derived from standard error (SE), confidence intervals (CIs), or p values using standard formulae. For cross-over studies, only pre-cross-over data were extracted; multi-arm trials were extracted by arm. We preferentially used intention-to-treat denominators for response/remission when available; otherwise, available-case data were extracted as reported.

### Quality assessment

Risk of bias was assessed using the Cochrane Risk of Bias tool [17]. Two reviewers independently rated each domain as low, unclear, or high risk; disagreements were resolved by consensus or third-party adjudication. An overall risk-of-bias judgment was then assigned for each study.

### Statistical analyses

TMS dose was defined a priori across four dimensions: total pulses, pulses per session, number of sessions, and treatment duration in weeks (from the first to the last day of stimulation). For the clinical interpretation of dose-response curves, we pre-specified categorical dose levels (low, mid-range, high) for each dimension, based on established therapeutic ranges from previous studies [7, 8]. Mid-range dose was defined as follows: 30,000–40,000 total pulses; 1,500–2,000 pulses/session; 10–20 sessions; and 2–3 weeks’ duration. Doses below and above these ranges were classified as low and high, respectively. Each dimension was analyzed separately in univariable dose-response models. Stimulation intensity, expressed as percentage of motor threshold where available, was extracted as an important protocol-level characteristic but was not prespecified as one of the four primary dose-response dimensions, because it reflects stimulation strength per pulse rather than cumulative or schedule-related exposure across the treatment course. The primary dose-response analyses therefore focused on total pulses, pulses per session, number of sessions, and treatment duration. Treatment effects were summarized as standardized mean differences (SMD) for continuous depressive-symptom scores and log risk ratios (logRR) for binary endpoints (response, remission), with 95% CIs. For binary data, logRRs and standard errors were computed from 2 × 2 tables; when any cell count was zero, the Haldane-Anscombe 0.5 continuity correction was applied [18]. Binary outcomes were modeled on the log-risk-ratio scale and are reported as risk ratios (RRs) (by exponentiation) with 95% CIs.

Dose-response relations were estimated using a random-effects model with the between-study variance estimated by restricted maximum likelihood (REML), implemented in *dosresmeta* [19]. Non-linearity was accommodated with restricted cubic splines (three knots at the 10th, 50th, and 90th centiles of the observed dose distribution) [20]. Wald χ² tests evaluated the overall association and departure from linearity. The maximum effective dose (EDmax), defined as the dose associated with the highest predicted benefit within the observed range for each dosing dimension considered separately, was estimated from the fitted spline. EDmax estimates were interpreted as univariable, group-level dose-response landmarks rather than as components of a combined optimal treatment protocol. Pointwise 95% confidence intervals were obtained using the delta method.

We prespecified a tiered approach for sensitivity analysis. When both the overall dose-response and the non-linearity test were significant, we assessed robustness of the EDmax with leave-one-out re-fits of the spline model, recording interior peaks only [19, 21]. When the overall association was significant but non-linearity was not, we focused on the linear dose trend: a random-effects meta-regression [22, 23]. We summarized leave-one-out slopes (median, interquartile range, sign reversals, and loss of significance) and compared the linear and spline specifications using the Akaike Information Criterion and a Wald test for non-linearity. Publication bias and small-study effects were examined using contour-enhanced funnel plots and dose-adjusted Egger-type meta-regressions [24]. We additionally conducted prespecified subgroup analyses by stimulation modality, with conventional rTMS and TBS examined separately across total pulses, pulses per session, total sessions, and treatment duration. We also performed a diagnosis-restricted subgroup analysis restricted to unipolar depression to assess the robustness of the main findings after exclusion of bipolar and mixed diagnostic populations. Two-sided *p* < 0.05 was considered statistically significant. Analyses were conducted in R (version 4.4.3).

## Results

### Study characteristics

**Fig. 1:**
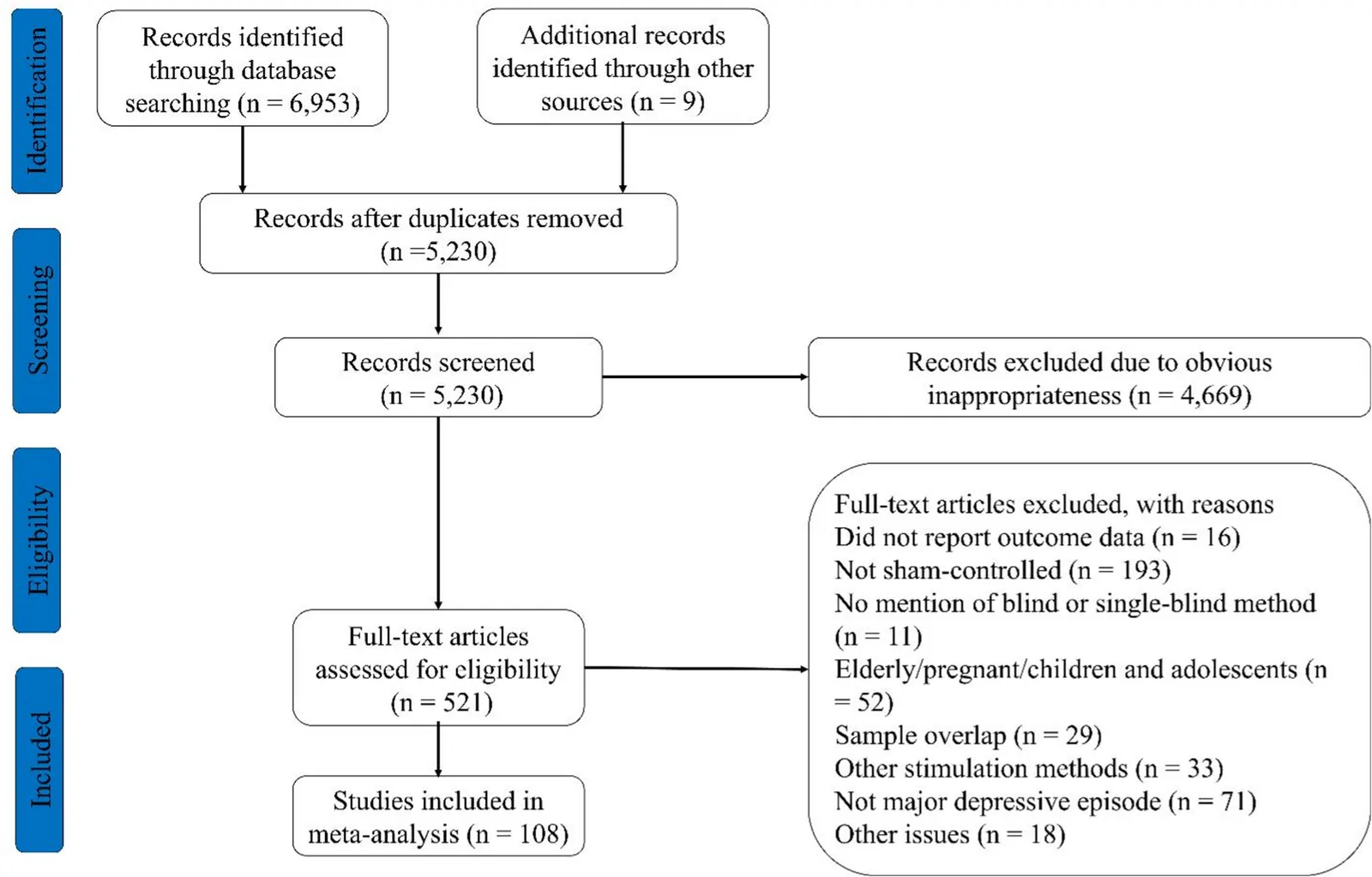
Flowchart of included studies

We retrieved 6,962 published records in all three literature databases, of which 108 RCTs with sham arms met inclusion criteria, comprising 134 intervention arms (Fig. 1) [25–132]. In total, 5,621 participants were enrolled: 3,021 randomized to active rTMS (mean age 43.3 years; 59.2% female) and 2,590 to sham (mean age 42.5 years; 55.8% female). Among the included trials, 38 focused on MDD [25, 29, 41, 45, 46, 49–53, 64, 67, 69, 71, 77, 79, 80, 82, 84, 90–92, 96, 97, 101, 103, 110, 111, 114, 118, 119, 121–123, 125, 126, 129, 130], 12 on bipolar disorder (BD) [30, 32, 37, 48, 56, 68, 72, 75, 85, 105, 106, 117], and 16 included mixed diagnostic populations [27, 34, 40, 43, 47, 55, 65, 81, 88, 89, 95, 100, 115, 120, 124, 127]. Separately, 42 trials enrolled TRD samples [26, 28, 31, 33, 35–39, 42, 44, 54, 57–63, 66, 70, 73, 74, 76, 78, 83, 86, 87, 93, 94, 98, 99, 102, 104, 107–109, 112, 113, 116, 128, 131, 132]. Because diagnosis and treatment-resistance status were coded as separate variables, these classifications were not mutually exclusive. Full study characteristics and demographics are presented in Additional File 1: Table S1 [25–132] and Fig. S1.

Across the 134 active arms, 104 used conventional rTMS [25–36, 40–45, 47–50, 53–55, 57–62, 64–67, 69, 71–74, 76–81, 83–87, 89–91, 94, 96–105, 107, 108, 112–125, 128–132] and 30 applied TBS (iTBS and cTBS) [37–39, 45, 46, 50–52, 56, 57, 63, 68, 70, 75, 82, 88, 90, 92, 93, 95, 106, 109–111, 113, 119, 126, 127]. Most targeted the left dorsolateral prefrontal cortex (DLPFC; Additional File 1: Fig. S1A, 69.4%) [25–28, 31–33, 36–40, 42–45, 47, 49, 53, 54, 57–59, 62, 64–74, 77–85, 89–91, 94, 96–105, 107, 109–125, 128–130]. The most frequent parameters were 110% of resting motor threshold (MT; Additional File 1: Fig. S1B, 29.1%) [26, 30, 32, 35, 36, 39, 41–45, 47–50, 54–56, 73, 74, 76, 78, 85–87, 107, 108, 110, 111, 115–117, 122, 129–131], 10 Hz frequency (Additional File 1: Fig. S1C, 35.8%) [26, 28, 32, 33, 35, 42–45, 53, 54, 57, 58, 62, 64, 67, 69, 71–74, 77, 80, 81, 84, 90, 91, 96, 97, 99, 101–103, 105, 107, 113–115, 117, 119, 121, 122, 124, 132], 2-week treatment duration (Additional File 1: Fig. S1D, 45.5%) [25, 27, 31, 32, 34, 35, 40, 41, 44, 45, 49, 51–53, 55, 57, 59, 69, 70, 73, 76, 81–83, 85–87, 89, 90, 94, 95, 97, 99, 101, 104, 107, 108, 112, 113, 118, 119, 122, 124, 125], 10 total sessions (Additional File 1: Fig. S1E, 38.8%) [25, 27, 31, 32, 40, 41, 44, 45, 49, 51, 53, 55, 57, 59, 69, 70, 76, 81, 83, 85, 87, 89, 94, 95, 99, 101, 104, 107, 108, 112–114, 118, 122, 124, 125], 1,600 pulses per session (Additional File 1: Fig. S1F, 17.6%) [26, 27, 40, 44, 45, 47, 53, 55, 57, 81, 85, 107, 114, 122, 128], and 16,000 total pulses (Additional File 1: Fig. S1G, 13.9%, ranged widely from 1,200 to 160,000 pulses) [27, 40, 44, 45, 53, 55, 57, 81, 85, 107, 114, 122].

### Quality of evidence

Risk-of-bias assessments are summarized in Additional File 1: Table S2 and Fig. S1. Sequence generation was low risk in 65 trials (60.2%) and unclear in 43 (39.8%). Allocation concealment was low in 45 (41.7%), unclear in 60 (55.6%), and high in 3 (2.8%). Blinding of participants and personnel was low in 66 (61.1%) and unclear in 42 (38.9%). Blinding of outcome assessment was low in 103 (95.4%) and unclear in 5 (4.6%). Incomplete outcome data were low in 91 (84.3%), unclear in 11 (10.2%), and high in 6 (5.6%). Selective reporting was low in 104 (96.3%) and unclear in 4 (3.7%). Overall risk of bias was rated low in 54 trials (50.0%), unclear in 45 (41.7%), and high in 9 (8.3%).

### Dose-response meta-analysis for total pulses

**Fig. 2:**
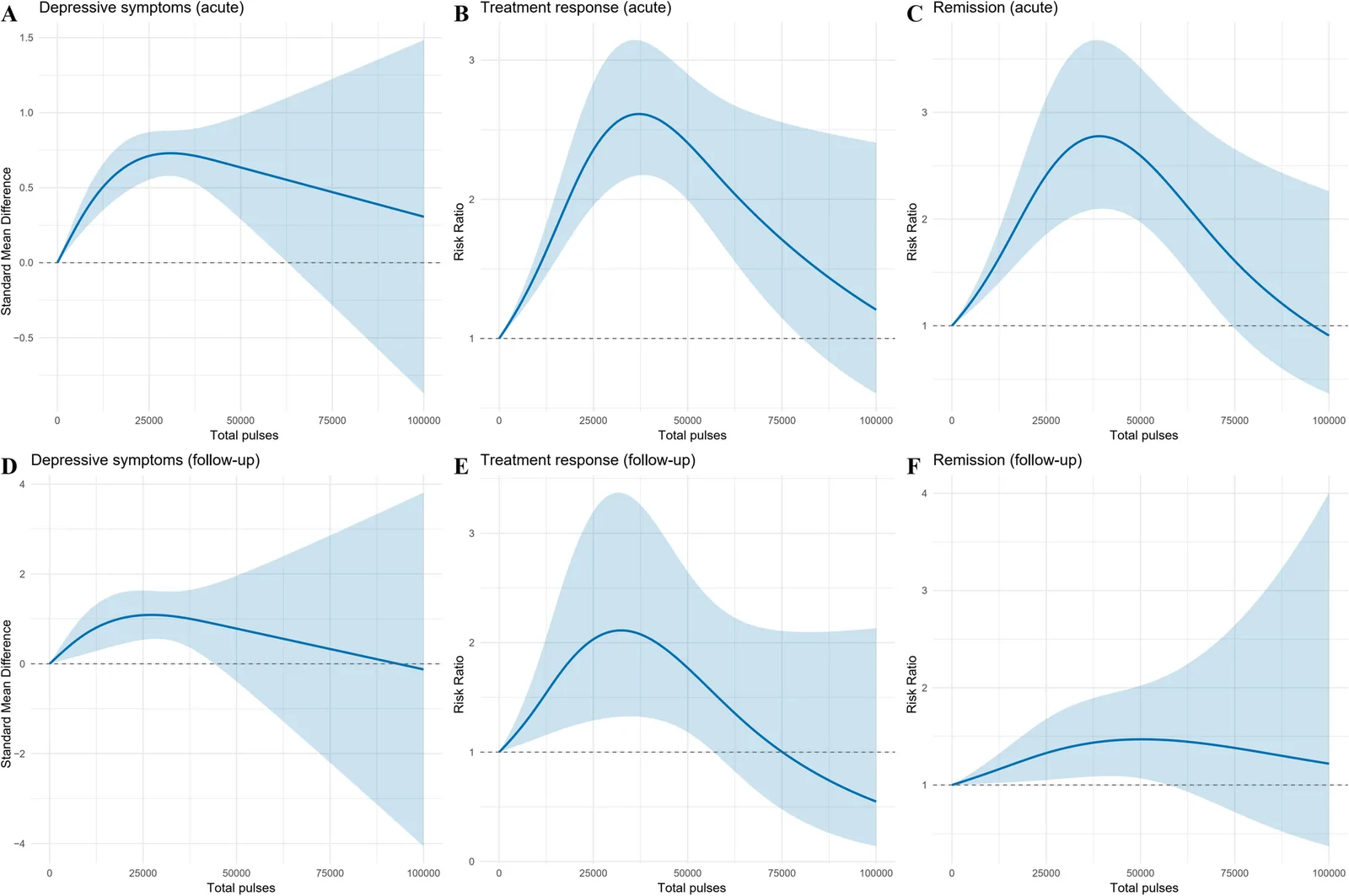
Dose-response associations of rTMS with total pulses across depressive-symptom, treatment-response, and remission outcomes. Panels (**A**), (**D**) display standardized mean differences (SMDs) for continuous depressive-symptom scores, and Panels (**B**), (**C**), (**E**), (**F**) display risk ratios (RR) for binary endpoints (treatment response and remission), during acute and follow-up periods. Curves were fitted using random-effects restricted cubic spline models with REML estimation. Shaded areas indicate pointwise 95% confidence intervals

At the group level, the dose-response associations for total pulses are shown in Fig. 2 and Additional File 1: Table S3. For depressive symptoms, the group-level dose-response pattern was significantly non-linear. In acute analyses (105 studies, 126 effect sizes), improvement peaked at 30,800 pulses (95% CI 16,100–45,600), with a predicted SMD of 0.73 (95% CI 0.58–0.88), after which the curve plateaued. At follow-up (30 studies; 35 effect sizes), the overall association remained significant but was approximately linear across the observed range, with no evidence of a distinct peak. For treatment response, the acute effect (87 studies, 100 effect sizes) peaked at 37,000 pulses (95% CI 30,700–43,400), with a predicted RR of 2.61 (95% CI 2.18–3.16), and decreased thereafter. At follow-up (14 studies, 16 effect sizes), the optimum was 32,300 pulses (95% CI 25,000–39,700), with a predicted RR of 2.12 (95% CI 1.32–3.35), but effects diminished at higher exposures. For remission, the acute optimum (58 studies, 72 effect sizes) was 39,100 pulses (95% CI 32,000–46,100), with a predicted RR of 2.77 (95% CI 2.10–3.67), followed by gradual decline. At follow-up (11 studies, 11 effect sizes), the curve was flatter, with no clear peak.

### Dose-response meta-analysis for pulses per session

**Fig. 3:**
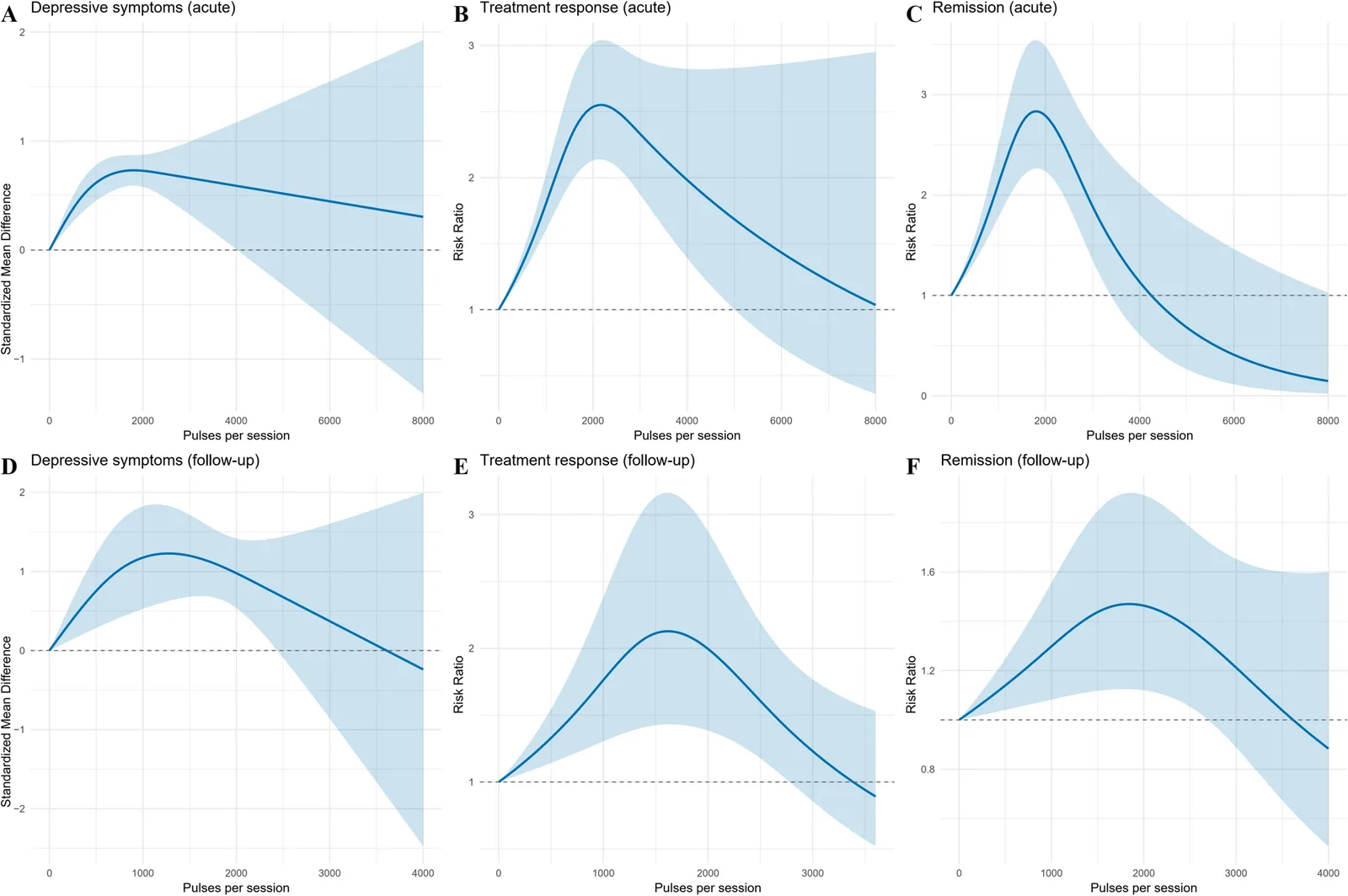
Dose-response associations of rTMS with pulses per session across depressive-symptom, treatment-response, and remission outcomes. Panels (**A**), (**D**) display standardized mean differences (SMDs) for continuous depressive-symptom scores, and Panels (**B**), (**C**), (**E**), (**F**) display risk ratios (RR) for binary endpoints (treatment response and remission), during acute and follow-up periods. Curves were fitted using random-effects restricted cubic spline models with REML estimation. Shaded areas indicate pointwise 95% confidence intervals

At the group level, associations between pulses per session and outcomes are shown in Fig. 3 and Additional File 1: Table S4. In acute analyses, depressive symptoms indicated an optimum at 1,800 pulses/session (95% CI 870–2,740), with a predicted SMD of 0.73 (95% CI 0.59–0.87), then gradually declined. At follow-up, the optimum was 1,300 pulses/session (95% CI 870–1,680), with a predicted SMD of 1.23 (95% CI 0.63–1.83), followed by decline. Regarding treatment response, the acute effect peaked at 2,200 pulses/session (95% CI 1,830–2,510), with a predicted RR of 2.56 (95% CI 2.14–3.03), then decreased. At follow-up, the optimum was 1,600 pulses/session (95% CI 1,480–1,760), with a predicted RR of 2.12 (95% CI 1.43–3.16), again followed by decline. Similarly, for remission, the acute peak was 1,800 pulses/session (95% CI 1,610–2,000), with a predicted RR of 2.83 (95% CI 2.27–3.56), after which the effect tapered. At follow-up, the maximum was 1,840 pulses/session (95% CI 1,490–2,190), with a predicted RR of 1.48 (95% CI 1.13–1.92).

### Dose-response meta-analysis for total sessions

**Fig. 4:**
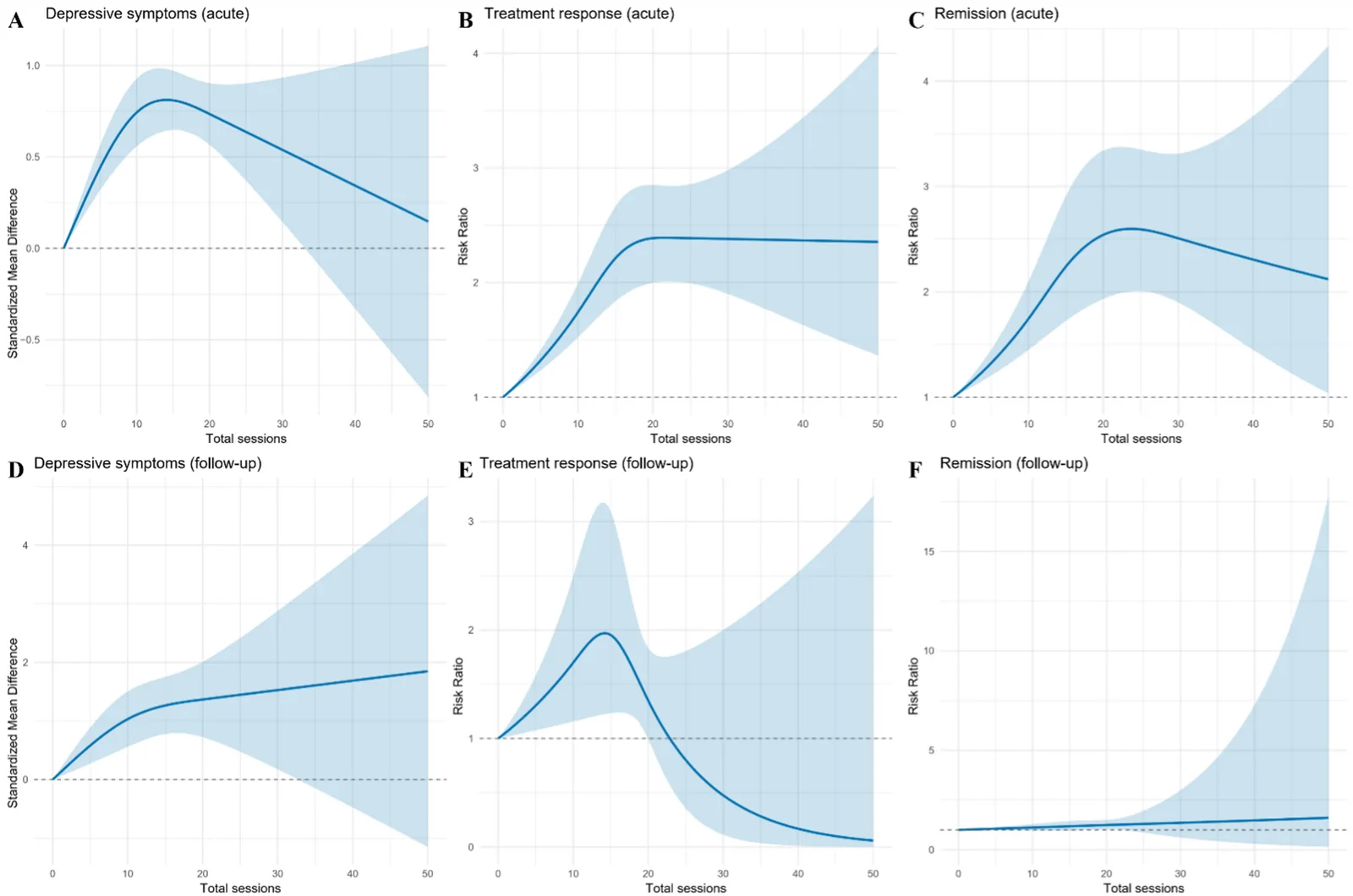
Dose-response associations of rTMS with total sessions across depressive-symptom, treatment-response, and remission outcomes. Panels (**A**), (**D**) display standardized mean differences (SMDs) for continuous depressive-symptom scores, and Panels (**B**), (**C**), (**E**), (**F**) display risk ratios (RR) for binary endpoints (treatment response and remission), during acute and follow-up periods. Curves were fitted using random-effects restricted cubic spline models with REML estimation. Shaded areas indicate pointwise 95% confidence intervals.

The associations for total number of sessions are shown in Fig. 4 and Additional File 1: Table S5. In terms of depressive symptoms, acute analyses peaked at 14 sessions (95% CI 11–17), with a predicted SMD of 0.81 (95% CI 0.64–0.98), followed by decline. At follow-up, the association appeared linear across the examined range, with no indication of a distinct peak. For treatment response, the acute optimum was 16 sessions (95% CI 12–19), with a predicted RR of 2.29 (95% CI 1.90–2.77). At follow-up, the dose–response relation remained broadly linear without evidence of a peak or downturn. Regarding remission, acute effects peaked at 23 sessions (95% CI 17–29), with a predicted RR of 2.59 (95% CI 1.99–3.39), followed by gradual decline. At follow-up, no clear maximum was identified; the overall association was not statistically significant (χ²=5.09, df = 2; *p* = 0.078).

### Dose-response meta-analysis for treatment duration

**Fig. 5:**
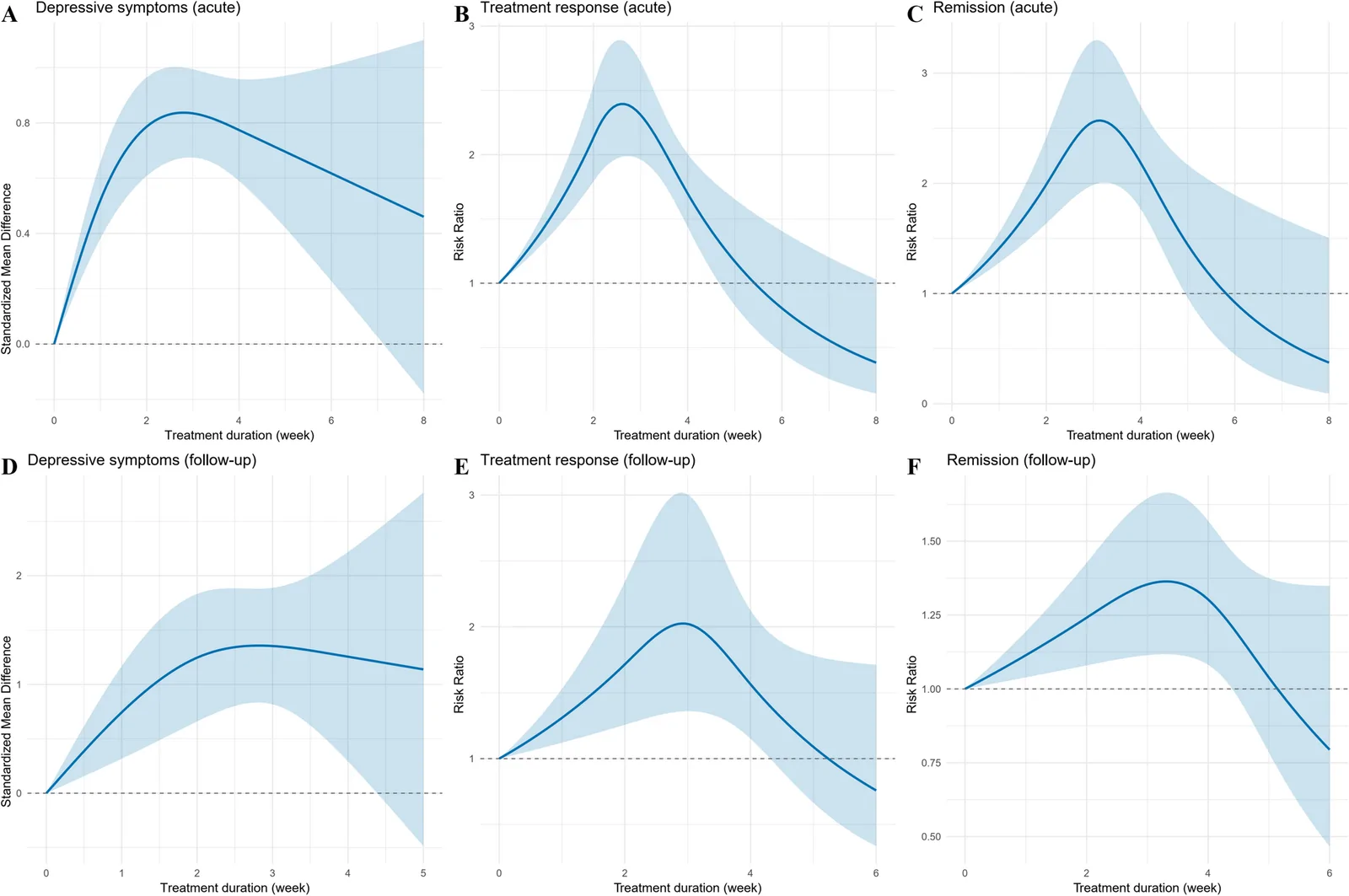
Dose-response associations of rTMS with treatment duration (week) across depressive-symptom, treatment-response, and remission outcomes. Panels (**A**), (**D**) display standardized mean differences (SMDs) for continuous depressive-symptom scores, and Panels (**B**), (**C**), (**E**), (**F**) display risk ratios (RR) for binary endpoints (treatment response and remission), during acute and follow-up periods. Curves were fitted using random-effects restricted cubic spline models with REML estimation. Shaded areas indicate pointwise 95% confidence intervals

The associations between treatment duration and outcomes are shown in Fig. 5 and Additional File 1: Table S6. Analysis of acute depressive symptoms showed a peak at 2.9 weeks (95% CI 2.4–3.5), with a predicted SMD of 0.84 (95% CI 0.67–1.00). At follow-up, the test for non-linearity was not significant. Acute treatment response rose to a peak at 2.7 weeks (95% CI 2.2–3.2; predicted RR 2.39, 95% CI 1.99–2.89). This outcome at follow-up was maximized at 2.9 weeks (95% CI 2.4–3.5; predicted RR 2.03, 95% CI 1.36–3.00), after which it declined. Finally, the optimum for acute remission was 3.1 weeks (95% CI 2.5–3.7; predicted RR 2.56, 95% CI 2.01–3.32), while the follow-up maximum was 3.3 weeks (95% CI 2.6–4.0; predicted RR 1.36, 95% CI 1.12–1.66), with no further gains beyond this point.

### Subgroup analysis for rTMS and TBS

Eight prespecified subgroup models were examined (rTMS and TBS each assessed for total pulses, pulses per session, total sessions, and treatment duration). For conventional rTMS, acute outcomes consistently demonstrated non-linear associations in separate univariable subgroup models, with the highest predicted group-level benefits observed within moderate exposure ranges for each dosing dimension separately: 30,000 to 40,000 total pulses, 1,500 to 2,300 pulses per session, 15 to 17 sessions, and about 3 weeks of treatment. At follow-up, effects were weaker and largely linear; where peaks appeared, they were small and exploratory. For TBS, acute outcomes showed similar patterns but at lower dose levels, with optima around 2,000 pulses per session, 8 to 10 sessions, and 2 to 2.5 weeks of treatment. Total pulse analyses suggested peaks near 40,000 for response, but follow-up evidence was inconsistent, with sparse data (≤ 5 studies in several strata) yielding unstable curves and wide uncertainty.

Taken together, the subgroup analyses indicate that, at the group level, moderate exposure ranges in separate univariable models, rather than the highest exposures, are associated with the greatest acute benefit for both rTMS and TBS. Follow-up effects were smaller, often linear, and less precisely estimated, especially for TBS. We therefore highlight acute non-linearity and mid-range plateaus as the most reproducible findings, while treating apparent follow-up peaks as hypothesis-generating given limited data. Full subgroup outputs are reported in Additional File 1: Tables S7–S14 and Figs. S3–S10.

### Subgroup analysis for unipolar depression

In subgroup analyses restricted to unipolar depression, the overall dose-response patterns were highly consistent with those of the primary analyses. Across all four dosing dimensions, acute outcomes continued to show non-linear associations in separate univariable models, with the highest predicted group-level benefits observed within moderate ranges for each dosing dimension separately: approximately 41,000 to 46,000 total pulses, 1,900 to 2,300 pulses per session, 14 to 22 sessions, and about 2.7 to 3.2 weeks of treatment. At follow-up, effects were generally weaker and less stable, with fewer studies and less consistent peaks, particularly for remission outcomes. Overall, the estimated optimal treatment duration remained centered at approximately 3 weeks, and the optimal range for pulses per session remained broadly similar to that observed in the full sample. Some differences were noted in EDmax estimates, with modest rightward shifts for total pulses across acute outcomes and for total sessions in acute response compared with the main analyses. However, these differences were small and did not materially alter the overall interpretation of the findings. Full subgroup outputs are reported in Additional File 1: Tables S15–S18 and Figs. S11–S14.

### Sensitivity analyses and publication bias

The robustness of our dose-response findings was supported by leave-one-out sensitivity analyses, which demonstrated stable EDmax estimates and linear slopes without sign reversals. Publication bias, evaluated using Egger’s test, was generally absent. Significant small-study effects were observed only in a few outcomes, particularly those assessing follow-up efficacy. Comprehensive results for these sensitivity and publication bias analyses are presented in the Additional File 1 (see the Sensitivity analysis section), including detailed summaries (Additional File 1: Table S19) and supporting visualizations (leave-one-out analyses in Additional File 1: Figs. S15–S18; funnel plots in Figs. S19–S22).

## Discussion

In this dose-response meta-analysis of 108 randomized, sham-controlled clinical trials, group-level dose-response models showed consistent non-linear associations between four rTMS dosing parameters (total pulses, pulses per session, number of sessions, and treatment duration) and acute outcomes in MDE [7, 8, 10]. Across continuous and binary endpoints, benefits generally peaked at mid-range exposures and then plateaued or declined, indicating that more stimulation is not invariably better. For conventional rTMS, the dose ranges associated with the highest predicted acute benefit in separate univariable models were 30,000–40,000 total pulses, 1,500–2,300 pulses per session, 15–17 sessions, and approximately 3 weeks of treatment. Follow-up effects were smaller, often near-linear, and imprecisely estimated. TBS showed a qualitatively similar pattern, with optima at lower exposures of approximately 2 000 pulses per session, 8–10 sessions, and about 2–2.5 weeks of treatment. However, the evidence base was relatively sparse, particularly for follow-up outcomes. These values should be interpreted as separate group-level dose-response landmarks rather than as a single combined optimal protocol.

A key observation is that estimated EDmax values differ across depressive-symptom change (continuous), response (≥ 50% reduction), and remission (scale-defined thresholds). This divergence is expected and clinically informative for three reasons. First, the endpoints capture progressively stricter clinical goals: continuous scores capture broad improvements; response requires surpassing a relative threshold, and remission requires crossing an absolute low-symptom boundary. As the targets become more stringent, the dose-response curves may shift rightward or flatten because additional “consolidation” of benefit is needed to convert partial improvement into threshold-crossing events. Second, the endpoints are modeled on different statistical scales. Continuous outcomes summarize mean change on an approximately linear scale, whereas response and remission are estimated from binary outcomes and therefore tend to flatten as probabilities approach their upper bounds. Third, the four dosing parameters capture different aspects of treatment exposure. Pulses per session mainly reflect within-session induction, whereas the number of sessions and treatment duration more closely reflect between-session consolidation. Total pulses combine these components and can be reached through heterogeneous schedules with distinct neuroplastic and tolerability profiles. Accordingly, endpoint-specific EDmax values should be interpreted as complementary rather than prescriptive, especially given the overlapping confidence intervals.

These findings have clear clinical relevance, but should be interpreted as group-level reference points rather than treatment rules. the univariable dose-response curves suggest that much of the acute benefit is achieved within moderate exposure ranges [133, 134]. By contrast, achieving response or remission seems to depend more on adequate consolidation through sufficient sessions and treatment duration, although gains diminish at higher exposures. This pattern is consistent with metaplastic counter-regulation [135] and with practical limits on adherence and tolerability observed in real-world settings [136, 137]. The modest and largely linear associations observed at follow-up suggest that sustaining treatment effects may require strategies beyond simple dose escalation. In particular, because the acute dose-response curves generally plateaued within moderate exposure ranges, further increases in session number alone would not be expected to yield proportional additional benefit once an adequate acute course has been delivered. In this context, maintaining benefit may depend more on consolidation, continuation, or maintenance strategies than on extending acute-session exposure alone. These may include continuation or maintenance TMS [138, 139], optimization of pharmacotherapy [140], and psychotherapy [141] tailored to individual risk profiles and early treatment response. Notably, pulse-related estimates at follow-up were often lower than those associated with the greatest acute benefit, although this pattern was not uniform across all models. This may partly reflect the smaller and less stable follow-up evidence base, especially at the upper end of the dose range. It may also suggest that the exposure needed to maintain benefit after treatment differs from that required for initial induction of antidepressant effects.

These group-level findings may also be useful in thinking through several practical questions, although they do not provide individual-level treatment rules. For example, the rightward shift of some response and remission curves suggests that threshold-based outcomes may require more consolidation than early symptom reduction, which could be particularly relevant in patients with greater baseline severity. At the same time, baseline severity was not modelled directly as a covariate in the present analyses, and treatment-resistance status was also not incorporated formally into the dose-response models. These interpretations should therefore remain cautious and should not be taken as individualized treatment rules. The results also do not support a simple view that accelerated protocols should always involve higher total pulse counts than non-accelerated protocols. Instead, treatment schedule, tolerability, and consolidation likely matter alongside total exposure. Similarly, although iTBS and conventional rTMS showed broadly similar qualitative patterns, the evidence base for TBS, especially for follow-up outcomes, remains more limited, so protocol-specific conclusions should be drawn cautiously. Finally, the observed plateaus suggest that extending treatment far beyond moderate exposure levels is unlikely to yield proportional additional benefit on average. When improvement remains limited after an adequate course, it may be more appropriate to reconsider the treatment strategy than to continue escalating dose alone.

Beyond these clinical implications, the present study adds to the literature in several methodological respects. Previous dose-response meta-analyses have typically focused on a single exposure metric, most often total pulses, or on a limited set of outcomes [7, 8]. By contrast, our analysis examined four dosing dimensions separately—total pulses, pulses per session, number of sessions, and treatment duration—and linked each of them to both acute and follow-up outcomes across continuous depressive-symptom change and the binary clinical endpoints of response and remission. This design makes it possible to distinguish induction-related and consolidation-related aspects of treatment exposure, and to show that apparent EDmax values depend not only on the stimulation parameter itself but also on the outcome definition and time window being considered. In this sense, the current findings are intended less as fixed prescriptions than as a descriptive group-level map of where benefit tends to plateau across commonly used treatment goals. These estimates reflect separate univariable group-level dose-response patterns and should not be interpreted as defining individual-level optima or a combined optimal protocol for clinical decision-making.

Several mechanisms may underlie these plateaus. rTMS involves neural plasticity mechanisms regulated by homeostatic or metaplastic processes [142], and excessive or prolonged stimulation can trigger counter-regulatory responses that reduce overall benefit [143]. Clinically, higher stimulation doses can lead to greater fatigue and scalp discomfort, which may limit tolerability. Higher dropout rates have been reported in TMS programmes and can, in turn, reduce the effective dose delivered and overall adherence [144]. Greater pulses per session also tend to co-vary with other parameters, such as inter-train interval, frequency, and intensity, which may alter the balance between facilitatory and inhibitory effects [145]. Although these dose-response analyses cannot fully separate such co-variations, the consistent shape of the curves across endpoints argues against a purely statistical explanation.

Our primary analysis was designed to examine dose-response relationships across broadly defined major depressive episodes and therefore included both unipolar and bipolar depressive episodes. To address potential diagnostic heterogeneity and improve clinical interpretability, we additionally performed a sensitivity analysis restricted to unipolar depression. The resulting estimates were highly consistent with the primary findings, although minor differences were observed, suggesting that inclusion of bipolar and mixed diagnostic populations did not materially alter the overall pattern of results.

Several limitations should be acknowledged. Follow-up data, particularly for TBS, were limited, resulting in wide uncertainty at the extremes of the dose distribution. In addition, because most included trials lasted less than 4 weeks, data were relatively sparse at higher exposure levels, particularly for treatment duration, total sessions, and total pulses. Although the random-effects modelling approach allowed us to incorporate the full range of observed dose values, it could not fully compensate for limited data in the upper parameter space, and estimates in these regions should therefore be interpreted with caution.

Although random-effects spline models help reduce ecological bias and capture non-linear trends, each dose parameter was analyzed separately. Residual confounding by unmeasured factors such as stimulation frequency, target site, intensity, coil type, or navigation method therefore remains possible, and total pulses are mathematically dependent on the other components [146]. Although stimulation intensity is clinically important and is usually reported as a percentage of motor threshold [2, 3], it was not modelled as a primary dose-response dimension in the present analysis. This decision was not based on an assumption that motor-threshold-based intensity is incomparable across studies, but rather on the prespecified focus of this study on cumulative and schedule-related exposure parameters. Stimulation intensity reflects the strength of stimulation per pulse, whereas total pulses, pulses per session, number of sessions, and treatment duration describe the amount and temporal distribution of stimulation delivered across the treatment course. In addition, intensity was reported relative to related but not fully identical threshold definitions, mainly resting motor threshold and active motor threshold [2]. Baseline symptom severity and treatment-resistance status may also have influenced treatment response and the apparent location of dose-response optima, particularly for threshold-based outcomes such as response and remission, but they were not modelled directly as covariates in the present analyses.

In addition, the primary analyses pooled sham-controlled TMS protocols across different stimulation targets, frequencies, and protocol classes to maximize statistical power and dose-range coverage. Although this broad analytic strategy is consistent with prior TMS meta-analytic work [7–10, 147], it may also have obscured protocol-specific differences, particularly because paradigms may differ in neurobiological mechanism or clinical implementation. Potential interactions among dose dimensions, including total pulses, pulses per session, number of sessions, and treatment duration, were not directly modeled [148]. Therefore, the present analysis cannot identify the optimal combination of treatment parameters for an individual protocol. A multivariable approach might better capture how these parameters jointly shape treatment outcomes, but such modelling was not adopted here because the dose dimensions were strongly interrelated, some were mathematically dependent, and simultaneous modelling of multiple continuous non-linear effects would have substantially increased model complexity relative to the available number of studies, particularly for follow-up outcomes and binary endpoints such as response and remission [148]. These issues should be further explored in future research.

Most included trials targeted the left DLPFC using 10 Hz stimulation at about 110% of the motor threshold for 2–3 weeks of treatment. Generalizability to other stimulation targets remains limited, and target-specific efficacy differences may exist [66]. Future research could employ network meta-analysis to compare and rank target sites more systematically [5]. We also restricted the analysis to adults aged 18–65 years, which may limit generalizability to adolescent and older adult populations. This prespecified age range was retained to reduce age-related heterogeneity in the dose-response models, given that adolescent and geriatric rTMS populations are often considered methodologically distinct from the general adult literature [149, 150], and older adults may differ in clinically relevant factors such as cortical atrophy and related stimulation considerations [150]. In addition, age may moderate rTMS dose-response relationships [8]. Dedicated meta-analyses focused specifically on adolescent and older adult populations are needed to determine whether the present findings extend to these groups. Finally, variation in sham procedures, concomitant treatments, definitions of treatment-resistant depression, and rating scales also contributes to heterogeneity across studies [151].

## Conclusions

In summary, in separate univariable models, rTMS efficacy increased sharply at lower exposures, reached its greatest predicted group-level effect at moderate exposure ranges, and then plateaued or declined. The dose-response maxima varied across endpoints, reflecting differences in clinical definition, statistical scale, and the relative roles of induction and consolidation. These group-level findings provide descriptive group-level reference points for interpreting rTMS dosing, but should not be viewed as an individualized dosing rule or a single combined optimal protocol.

## Supplementary Information

Below is the link to the electronic supplementary material.

Supplementary Material 1: Additional file 1: Supplementary methods and Supplementary results, Tables S1–S19 and Figs. S1–S22.

Supplementary Material 2: PRISMA Checklist.

## Data Availability

The data used in this meta-analysis were extracted from previously published studies cited in the reference list. The data extraction and analytical code are available from the corresponding author upon reasonable request.

## Abbreviations

MDE: Major depressive episode
TMS: Transcranial magnetic stimulation
TRD: Treatment-resistant depression
PRISMA: Preferred Reporting Items for Systematic Reviews and Meta-Analyses
CENTRAL: Cochrane Central Register of Controlled Trials
RCTs: Randomized controlled trials
MDD: Major depressive disorder
DSM: Diagnostic and Statistical Manual of Mental Disorders
ICD: International Classification of Diseases
TBS: Theta-burst stimulation
iTBS: Intermittent theta-burst stimulation
cTBS: Continuous theta-burst stimulation
HAMD: Hamilton Rating Scale for Depression
MADRS: Montgomery-Asberg Depression Rating Scale
BDI: Beck Depression Inventory
SD: Standard deviation
MT: Motor threshold
SE: Standard error
Cis: Confidence intervals
SMD: Standardized mean differences
logRR: log risk ratios
RR: Risk ratios
REML: Restricted maximum likelihood
EDmax: Maximum effective dose
DLPFC: Dorsolateral prefrontal cortex
