# Supplementary material for: Acute and long-term effects of repetitive transcranial magnetic stimulation in major depressive episodes: a systematic review and dose-response meta-analysis of randomized sham-controlled trials
Source: BMC Med. 2026 Jul 30;24:622. doi: 10.1186/s12916-026-05089-z (PMC13746655; doi:10.1186/s12916-026-05089-z)
Supplement: Supplementary file 1 [file 12916_2026_5089_MOESM1_ESM.docx]

**Acute and long-term effects of repetitive transcranial magnetic stimulation in major depressive episodes: a systematic review and dose-response meta-analysis of randomized sham-controlled trials**

**Supplementary Material**

**Search strategy**

**Sensitivity analysis**

**Table S1**. Characteristics of included randomized sham-controlled trials in this study

**Table S2**. Quality assessment of the included articles.

**Table S3**. Dose-response associations of rTMS with total pulses for depressive symptoms, treatment response, and remission.

**Table S4**. Dose-response associations of rTMS with pulses per session for depressive symptoms, treatment response, and remission.

**Table S5**. Dose-response associations of rTMS with total sessions for depressive symptoms, treatment response, and remission.

**Table S6**. Dose-response associations of rTMS with treatment duration (week) for depressive symptoms, treatment response, and remission.

**Table S7**. Dose-response associations of rTMS subgroup with total pulses for depressive symptoms, treatment response, and remission.

**Table S8**. Dose-response associations of TBS subgroup with total pulses for depressive symptoms, treatment response, and remission.

**Table S9**. Dose-response associations of rTMS subgroup with pulses per session for depressive symptoms, treatment response, and remission.

**Table S10**. Dose-response associations of TBS subgroup with pulses per session for depressive symptoms, treatment response, and remission.

**Table S11**. Dose-response associations of rTMS subgroup with total sessions for depressive symptoms, treatment response, and remission.

**Table S12**. Dose-response associations of TBS subgroup with total sessions for depressive symptoms, treatment response, and remission.

**Table S13**. Dose-response associations of rTMS subgroup with treatment duration (week) for depressive symptoms, treatment response, and remission.

**Table S14**. Dose-response associations of TBS subgroup with treatment duration (week) for depressive symptoms, treatment response, and remission.

**Table S15** Dose-response associations of rTMS with total pulses in unipolar depression subgroup for depressive symptoms, treatment response, and remission.

**Table S16** Dose-response associations of rTMS with pulses per session in unipolar depression subgroup for depressive symptoms, treatment response, and remission.

**Table S17** Dose-response associations of rTMS with total sessions in unipolar depression subgroup for depressive symptoms, treatment response, and remission.

**Table S18** Dose-response associations of rTMS with total treatment duration (week) in unipolar depression subgroup for depressive symptoms, treatment response, and remission.

**Table S19**. Tests of heterogeneity and publication bias across rTMS dosing parameters and clinical outcomes.

**Fig. S1.** Overview of study characteristics and stimulation parameter distributions among 108 included randomized sham-controlled trials.

**Fig. S2**. Summary of risk of bias across the 108 included studies.

**Fig. S3**. Dose-response associations of rTMS subgroup with total pulses across depressive-symptom, treatment-response, and remission outcomes.

**Fig. S4**. Dose-response associations of rTMS subgroup with pulses per session across depressive-symptom, treatment-response, and remission outcomes.

**Fig. S5**. Dose-response associations of rTMS subgroup with total sessions across depressive-symptom, treatment-response, and remission outcomes.

**Fig. S6**. Dose-response associations of rTMS subgroup with treatment duration (week) across depressive-symptom, treatment-response, and remission outcomes.

**Fig. S7**. Dose-response associations of TBS subgroup with total pulses across depressive-symptom, treatment-response, and remission outcomes.

**Fig. S8**. Dose-response associations of TBS subgroup with pulses per session across depressive-symptom, treatment-response, and remission outcomes.

**Fig. S9**. Dose-response associations of TBS subgroup with total sessions across depressive-symptom, treatment-response, and remission outcomes.

**Fig. S10**. Dose-response associations of TBS subgroup with treatment duration (week) across depressive-symptom, treatment-response, and remission outcomes.

**Fig. S11**. Dose-response associations of unipolar depression subgroup with total pulses across depressive-symptom, treatment-response, and remission outcomes.

**Fig. S12**. Dose-response associations of unipolar depression subgroup with pulses per session across depressive-symptom, treatment-response, and remission outcomes.

**Fig. S13**. Dose-response associations of unipolar depression subgroup with total sessions across depressive-symptom, treatment-response, and remission outcomes.

**Fig. S14**. Dose-response associations of unipolar depression subgroup with treatment duration (week) across depressive-symptom, treatment-response, and remission outcomes.

**Fig. S15**. Leave-one-out sensitivity analyses for total-pulse-based dose-response models of rTMS.

**Fig. S16.** Leave-one-out sensitivity analyses for pulses-per-session–based dose–response models of rTMS.

**Fig. S17**. Leave-one-out sensitivity analyses for total-sessions–based dose–response models of rTMS.

**Fig. S18**. Leave-one-out sensitivity analyses for treatment-duration–based dose–response models of rTMS.

**Fig. S19**. Funnel plots of publication bias for total-pulse–based rTMS dose-response analyses across outcomes.

**Fig. S20**. Funnel plots of publication bias for pulse-per-session–based rTMS dose-response analyses across outcomes.

**Fig. S21**. Funnel plots of publication bias for total-sessions–based rTMS dose-response analyses across outcomes.

**Fig. S22**. Funnel plots of publication bias for treatment-duration–based rTMS dose-response analyses across outcomes.

**Search strategy**

**CENTRAL** - Cochrane Central Register of Controlled Trials: 1310

#1 "depression" OR "depressive disorder" OR "major depressive disorder" OR "unipolar depression" OR "depressive symptom*"

#2 "transcranial magnetic stimulation" OR TMS OR rTMS OR "magnetic stimulation"

#3 random* OR trial* OR sham

#4 #1 AND #2 AND #3

**Embase**: 5076

#1'depression'/exp OR 'major depression'/exp OR 'depressive disorder'/exp OR ("major depressive disorder":ti,ab OR "depressive disorder":ti,ab OR depression:ti,ab OR depressive:ti,ab OR "unipolar depression":ti,ab OR "depressive symptom*":ti,ab)

#2 'transcranial magnetic stimulation'/exp OR 'transcranial magnetic stimulation':ti,ab OR tms:ti,ab OR rtms:ti,ab OR 'magnetic stimulation':ti,ab

#3 'randomized controlled trial'/exp OR 'clinical trial'/exp OR random*:ti,ab OR trial*:ti,ab OR placebo:ti,ab OR sham:ti,ab

#4 #1 AND #2 AND #3

**PubMed**: 567

#1 ("depressive disorder"[MeSH Terms]) OR (“Depression” [Mesh terms]) OR ("Major Depressive Disorder"[Title/Abstract]) OR ("Depressive Disorder"[Title/Abstract]) OR ("Depressive"[Title/Abstract]) OR ("Unipolar Depression"[Title/Abstract]) OR ("Depression"[Title/Abstract]) OR ("Depressive Symptom"[Title/Abstract])

#2 ("Transcranial Magnetic Stimulation"[MeSH Terms]) OR ("Magnetic Stimulation "[Title/Abstract]) OR ("Transcranial Magnetic Stimulation"[Title/Abstract])

#3 ("Randomized Controlled Trial"[Publication Type] OR "randomized controlled trial"[Title/Abstract] OR "randomised controlled trial"[Title/Abstract] OR "RCT"[Title/Abstract] OR "random allocation"[Title/Abstract] OR "randomly assigned"[Title/Abstract])

#4 #1 AND #2 AND #3

**Sensitivity analysis**

**Total pulses**

**Acute depressive symptom severity**. Because both the overall and non-linearity tests were significant, we assessed the robustness of the maximum effective dose (EDmax). Leave-one-out analyses showed a highly stable interior maximum: the full-sample EDmax was approximately 30,800 pulses; across 105 refits, EDmax clustered near 30,800 (IQR 30,500–31,300). Only 1 of 105 refits (0.95%) showed no interior maximum (that is, a monotonic increase or plateau within the observed range), supporting the stability of the mid-to-high-dose peak and subsequent plateau.

**Follow-up depressive symptom severity**. The overall association was significant but the test for non-linearity was not. Sensitivity analyses therefore focused on the linear slope and model specification. Leave-one-out refits yielded a stable slope (median 0.645 SMD per 10,000 pulses; IQR 0.631–0.653) with no sign reversals and no loss of significance. Model comparison modestly favoured the linear over the spline form (AIC linear = 208.5 vs AIC spline = 211.1; ΔAIC = 2.5).

**Acute response**. With significant overall and non-linearity tests, EDmax stability was examined. The full-sample curve showed a clear interior EDmax at approximately 37,400 pulses. Leave-one-out refits produced virtually identical optima (median 37,400; IQR 37,400–37,800 pulses), and all refits retained an interior peak (0% without a peak), indicating a highly robust optimum.

**Follow-up response**. With significant overall and non-linearity tests in the log-risk-ratio model, the full-sample curve showed an interior maximum at approximately 32,400 pulses. Leave-one-out refits yielded closely similar optima (median 32,500; IQR 31,800–32,900 pulses), and all refits preserved an interior peak, indicating robustness to exclusion of any single trial.

**Acute remission**. Both overall and non-linearity tests were significant. The full-sample EDmax occurred at approximately 39,000 pulses. Leave-one-out analyses returned identical optima across all refits (median 39,000; IQR 37,020–40,020 pulses), with no loss of the interior peak, again supporting a highly robust optimum.

**Follow-up remission**. The overall association was significant whereas the non-linearity test was not; sensitivity analyses therefore targeted slope robustness and model choice. Leave-one-out slopes were stable (median 0.065 change in log(RR) per 10,000 pulses; IQR 0.059–0.073) with no sign reversals, though some refits lost significance at alpha = 0.05. Despite a Wald test suggesting non-linearity (chi-square = 6.60, df = 2; p = 0.0369), model fit strongly favoured a linear specification (AIC linear = 44.0 vs AIC spline = 67.1; ΔAIC = 23.1).

**Pulses per session**

**Acute depressive symptom severity.** The full-sample curve showed a clear interior EDmax at 1,804.5 pulses per session. Leave-one-out refits yielded virtually the same optimum (median 1,804.5; IQR 1,784.5–1,824.6). Only about 2 percent of refits (2 of 105) lost the interior peak, indicating that the location of the acute optimum is highly robust to exclusion of any single study.

**Follow-up depressive symptom severity.** The full-sample curve had an interior EDmax at 1,273.2 pulses per session. Leave-one-out refits gave nearly identical optima (median 1,268.2; IQR 1,253.1–1,303.3), and every refit retained an interior peak (0 percent without a peak). These results indicate a highly robust follow-up optimum.

**Acute response.** The full-sample curve showed an interior EDmax at 2,173.9 pulses per session. Leave-one-out refits yielded identical optima (median 2,173.9; IQR 2,173.9–2,173.9), and every refit retained an interior peak (0 percent without a peak), indicating that the acute-response optimum is exceptionally robust to the exclusion of any single study.

**Follow-up response.** The full-sample curve peaked at an interior EDmax of 1,615 pulses per session. Leave-one-out refits produced virtually identical optima (median 1,615; IQR 1,615–1,621.2), and all refits retained an interior peak (0 percent without a peak), indicating a highly robust follow-up response optimum.

**Acute remission.** The full-sample curve peaked at an interior EDmax of 1,799 pulses per session. Leave-one-out refits yielded identical optima (median 1,799; IQR 1,799–1,799), and all refits retained an interior peak (0 percent without a peak), indicating an exceptionally robust acute remission optimum.

**Follow-up remission.** The full-sample curve peaked at an interior EDmax of 1,839 pulses per session. Leave-one-out refits yielded near-identical optima (median 1,843; IQR 1,821–1,849), and every refit retained an interior peak (0 percent without a peak), indicating a highly robust follow-up remission optimum.

**Total sessions**

**Acute depressive symptom severity**. The full-sample curve peaked at an interior EDmax of 14 sessions. Leave-one-out refits produced virtually identical optima (median 14; IQR 14–14), and every refit retained an interior peak (0 percent without a peak), indicating a highly robust session-level optimum for acute symptom improvement.

**Follow-up depressive-symptom severity**. The overall association was significant while the test for non-linearity was not. Sensitivity analyses therefore targeted the linear slope and model form. Leave-one-out refits showed a stable slope of 0.086 SMD per additional session (median 0.087; IQR 0.085–0.089) with no sign reversals and no loss of significance. Model comparison favored the linear over the spline specification (AIC linear 155.6 vs AIC spline 162.5; ΔAIC 6.8).

**Acute response.** The dose–response curve showed a clear interior optimum at 16 sessions; leave-one-out refits yielded the same optimum (median 16; IQR 11–20). Most refits retained an interior peak, although 16.1% became monotonic within the observed range, indicating that the peak is generally robust but exhibits some sensitivity to the exclusion of individual trials.

**Follow-up response**. The overall association was significant while the test for non-linearity was not. Sensitivity analyses therefore targeted the linear slope and model form. Leave-one-out refits showed a stable slope of 0.0307 in log(RR) per additional session (median 0.0307; IQR 0.0246–0.0331) with no sign reversals and no loss of significance; on the RR scale, this is ≈3.1% higher response per extra session. Model comparison favored the linear over the spline specification (AIC linear 45.2 vs AIC spline 53.3; ΔAIC 8.1).

**Acute remission**. The dose–response curve showed a clear interior optimum at 23 sessions. Leave-one-out analyses yielded virtually identical optima (median 23; IQR 23–23 sessions), and every refit retained an interior peak (0% without a peak), indicating that the estimated EDmax is highly robust to exclusion of any single study.

**Follow-up remission.** This model was not significant, and no sensitivity analysis was conducted**.**

**Treatment duration**

**Acute depressive-symptom severity**. The dose–response curve showed a clear interior optimum at 2.9 weeks. Leave-one-out refits were identical (median 2.9; IQR 2.8–2.9 weeks), and every refit retained an interior peak (0% without), indicating the estimated EDmax is highly robust.

**Follow-up depressive-symptom severity**. The overall association was significant while the test for non-linearity was not. Sensitivity analyses therefore emphasized model form and slope stability. Leave-one-out refits showed a stable positive slope of 0.475 SMD per additional week (median 0.484; IQR 0.470–0.490) with no sign reversals and no loss of significance. Model comparison did not clearly favor either specification (AIC linear 164.9 vs AIC spline 164.6; ΔAIC −0.2).

**Acute response**. With a significant overall association and non-linearity, sensitivity analyses targeted EDmax. The full-sample curve peaked at 2.8 weeks; leave-one-out refits produced identical optima (median 2.8; IQR 2.7–2.9 weeks), and no refit lost the interior peak (0%). This indicates a highly robust short-duration optimum.

**Follow-up response**. With a significant overall association and non-linearity, sensitivity analyses focused on EDmax. The full-sample optimum was 2.9 weeks; leave-one-out refits yielded identical optima (median 2.9; IQR 2.4–3.1 weeks), and no refit lost the interior peak (0%). This indicates a highly robust short-duration optimum.

**Acute remission**. With a significant overall association and non-linearity, sensitivity analyses targeted EDmax. The full-sample optimum was 3.1 weeks; leave-one-out refits gave identical optima (median 3.1; IQR 3.1–3.1 weeks), and 0% of refits lost the interior peak—indicating a highly robust short-duration optimum.

**Follow-up remission**. With a significant overall association and non-linearity, we examined EDmax robustness. The full-sample optimum was 3.3 weeks; leave-one-out refits yielded identical optima (median 3.3; IQR 3.3–3.3 weeks), and 0% of refits lost the interior peak—indicating a highly robust short-duration optimum.

**Table S1**. Characteristics of included randomized sham-controlled trials in this study.

| Author, year, country | Treatment (sham control method) | Diagnosis | Diagnostic criteria | Treatment resistant^1^ | Sample size (randomized/completed) | Sex (male/female) | Age (mean, SD) | Rating scale | Depression severity (mean, SD) | Medication status | Washout period | Stimulated Brain Region | Frequency (Hz) | Intensity (MT%) | Pulses per session (total sessions) | Total pulses | Treatment duration |
| --- | --- | --- | --- | --- | --- | --- | --- | --- | --- | --- | --- | --- | --- | --- | --- | --- | --- |
| Akpinar et al., 2022, Turkey^[1]^ | rTMS | MDD | DSM-V | Yes | 20/20 | 3/17 | 43.7 ± 14.2 | HAMD-17 | 20.0 ± 3.3 | Stable SNRIs | No | Left DLPFC | 10 Hz | 110% | 1,000 (20) | 20,000 | 2 weeks |
|  | Sham (rotation 45°) | MDD | DSM-V | Yes | 18/18 | 3/15 | 45.6 ± 7.8 | HAMD-17 | 20.5 ± 3.4 | Stable SNRIs | No |  |  |  |  |  |  |
| Anderson et al., 2007, UK^[2]^ | rTMS | MDD | DSM-IV | Yes | 13/11 | 6/7 | 48.0 ± 8.0 | MADRS | 26.7 ± 3.6 | Stable Psychotropic drugs | No | Left DLPFC | 10 Hz | 110% | 1,000 (12–18) | 12,000–18,000 | 4–6 weeks |
|  | Matched sham coil | MDD | DSM-IV | Yes | 16/14 | 7/9 | 46.0 ± 12.0 | MADRS | 27.7 ± 7.1 | Stable Psychotropic drugs | No |  |  |  |  |  |  |
| Armas-Castañeda et al., 2021, Mexico^[3]^ | rTMS | MDD | DSM-IV | No | 25/25 | 7/18 | 23.5 ± 5.3 | HAMD-21 | 26.8 ± 4.1 | Drug-free | Yes | Left DLPFC | 5 Hz | 110% | 1,500 (15) | 22,500 | 3 weeks |
|  | Matched sham coil | MDD | DSM-IV | No | 22/22 | 5/17 | 21.3 ± 2.4 | HAMD-21 | 29.4 ± 5.4 | Drug-free | Yes |  |  |  |  |  |  |
|  | rTMS | MDD | DSM-IV | No | 22/22 | 5/17 | 21.2 ± 3.3 | HAMD-21 | 27.2 ± 3.9 | Drug-free | Yes | Left DLPFC | 5 Hz | 110% | 1,500 (15) | 22,500 | 7 weeks |
|  | Matched sham coil | MDD | DSM-IV | No | 22/22 | 8/14 | 22.1 ± 3.9 | HAMD-21 | 27.7 ± 4.4 | Drug-free | Yes |  |  |  |  |  |  |
| Asgharian Asl & Vaghef, 2022, Iran^[4]^ | rTMS | MDD | DSM-IV | No | 14/14 | 0/14 | 36.3 ± 8.8 | BDI-II | 36.3 ± 8.3 | Stable Psychotropic drugs | No | Left DLPFC | 20 Hz | 85% | 2,500 (10) | 25,000 | 2 weeks |
|  | Sham (rotation 90°) | MDD | DSM-IV | No | 14/14 | 0/14 | 34.5 ± 9.5 | BDI-II | 37.4 ± 10.9 | Stable Psychotropic drugs | No |  |  |  |  |  |  |
| Avery et al., 2006, USA^[5]^ | rTMS | MDD | DSM-IV | Yes | 35/33 | 14/21 | 44.3 ± 10.3 | HAMD-17 | 23.5 ± 3.9 | Stable Psychotropic drugs | No | Left DLPFC | 10 Hz | 110% | 1,600 (15) | 24,000 | 3 weeks |
|  | Sham (rotation 90°) | MDD | DSM-IV | Yes | 33/30 | 17/16 | 44.2 ± 9.7 | HAMD-17 | 23.5 ± 2.9 | Stable Psychotropic drugs | No |  |  |  |  |  |  |
| Bakim et al., 2012, Turkey^[6]^ | rTMS | MDD | DSM-V | Yes | 12/12 | 2/10 | 38.7 ± 10.0 | HAMD-17 | 23.1 ± 3.6 | Stable SNRIs/SSRIs | No | Left DLPFC | 20 Hz | 80% | 800 (30) | 24,000 | 6 weeks |
|  | rTMS | MDD | DSM-V | Yes | 11/11 | 1/10 | 43.1 ± 8.2 | HAMD-17 | 24.1 ± 2.8 | Stable SNRIs/SSRIs | No | Left DLPFC | 20 Hz | 110% | 800 (30) | 24,000 | 6 weeks |
|  | Sham (rotation 45°) | MDD | DSM-V | Yes | 12/12 | 1//11 | 44.4 ± 10.2 | HAMD-17 | 25.6 ± 3.8 | Stable SNRIs/SSRIs | No |  |  |  |  |  |  |
| Bengtsson et al., 2023, Sweden^[7]^ | iTBS | MDD | DSM-IV | No | 25/25 | 11/14 | 27.0 ± 9.6 | MADRS-S | 29.2 ± 8.3 | Stable Psychotropic drugs | No | DMPFC | 50 Hz bursts at 5 Hz | 90% | 1,200 (20) | 24,000 | 2 weeks |
|  | Matched sham coil | MDD | DSM-IV | No | 24/24 | 10/14 | 27.0 ± 8.5 | MADRS-S | 30.1 ± 7.3 | Stable Psychotropic drugs | No |  |  |  |  |  |  |
| Bengtsson et al., 2023, Sweden^[8]^ | iTBS | MDD | DSM-IV | No | 25/24 | 11/14 | 27.0 ± 9.6 | MADRS-S | 30.0 ± 8.0 | Stable Psychotropic drugs | No | DMPFC | 50 Hz bursts at 5 Hz | 90% | 1,200 (20–30) | 24,000–36,000 | 2–3 weeks |
|  | Matched sham coil | MDD | DSM-IV | No | 24/23 | 10/14 | 27.0 ± 8.5 | MADRS-S | 30.0 ± 7.0 | Stable Psychotropic drugs | No |  |  |  |  |  |  |
| Berman et al., 2000, USA^[9]^ | rTMS | MDD | DSM-IV | Yes | 10/10 | 8/2 | 44.5 | HAMD-25 | 37.2 ± 2.0 | Drug-free | Yes | Left DLPFC | 20 Hz | 80% | 800 (10) | 8,000 | 2 weeks |
|  | Matched sham coil | MDD | DSM-IV | Yes | 10/10 | 7/3 | 41.5 | HAMD-25 | 37.2 ± 2.0 | Drug-free | Yes |  |  |  |  |  |  |
| Blumberger et al., 2012, Canada^[10]^ | rTMS | MDD | DSM-IV | Yes | 26/22 | 12/14 | 58.0 ± 12.5 | HAMD-17 | 25.1 ± 3.8 | Stable Psychotropic drugs | No | Bilateral DLPFC | Right 1 Hz; Left 10 Hz | Right: 100%, Left: 110% | 1,215 (15–30) | 18,225– 36,450 | 3–6 weeks |
|  | rTMS | MDD | DSM-IV | Yes | 22/12 | 10/12 | 48.9 ± 13.4 | HAMD-17 | 26.0 ± 3.3 | Stable Psychotropic drugs | No | Left DLPFC | 10 Hz | Right: 100%, Left: 110% | 1,450 (15–30) | 21,750– 43,500 | 3–6 weeks |
|  | Sham (rotation 90°) | MDD | DSM-IV | Yes | 20/15 | 6/14 | 45.8 ± 13.4 | HAMD-17 | 25.2 ± 2.8 | Stable Psychotropic drugs | No |  |  |  |  |  |  |
| Boutros et al., 2002, USA^[11]^ | rTMS | MDD | DSM-IV | Yes | 12/11 | 8/4 | 49.5 ± 8.0 | HAMD-25 | 34.4 ± 10.1 | Stable Psychotropic drugs | No | Left DLPFC | 20 Hz | 80% | 800 (10) | 8,000 | 2 weeks |
|  | Sham (rotation 90°) | MDD | DSM-IV | Yes | 9/7 | 8/1 | 52.0 ± 7.0 | HAMD-25 | 31.7 ± 4.9 | Stable Psychotropic drugs | No |  |  |  |  |  |  |
| Bretlau et al., 2008, Denmark^[12]^ | rTMS | MDD | DSM-IV | Yes | 25/22 | 7/15 | 53.1 ± 10.1 | HAMD-17 | 25.3 ± 3.0 | Escitalopram | Yes | Left DLPFC | 8 Hz | 90% | 1,280 (15) | 19,200 | 3 weeks |
|  | Matched sham coil | MDD | DSM-IV | Yes | 24/23 | 10/13 | 57.8 ± 10.0 | HAMD-17 | 24.8 ± 3.2 | Escitalopram | Yes |  |  |  |  |  |  |
| Buchholtz Hansen et al., 2004, Denmark^[13]^ | rTMS | Mixed | DSM-IV | No | 8/6 | 4/2 | 46.2 ± 14.8 | HAMD-17 | 25.2 ± 4.5 | Stable Psychotropic drugs | No | Left DLPFC | 20 Hz | 90% | 2,000 (15) | 30,000 | 3 weeks |
|  | Sham (rotation 90°) | Mixed | DSM-IV | No | 7/7 | 5/2 | 50.7 ± 13.3 | HAMD-17 | 23.7 ± 6.4 | Stable Psychotropic drugs | No |  |  |  |  |  |  |
| Carpenter et al., 2017, USA^[14]^ | rTMS | MDD | DSM-IV | Yes | 47/38 | 13/34 | 45.6 ± 12.0 | HAMD-24 | 31.3 ± 5.5 | Stable antidepressants | No | Left DLPFC | 10 Hz | 120% | 3,000 (20) | 60,000 | 4 weeks |
|  | Matched sham coil | MDD | DSM-IV | Yes | 45/37 | 10/35 | 47.6 ± 13.1 | HAMD-24 | 31.1 ± 6.8 | Stable antidepressants | No |  |  |  |  |  |  |
| Chen et al., 2013, China^[15]^ | rTMS | MDD | DSM-IV | Yes | 10/10 | 3/7 | 44.1 ± 4.4 | HAMD-17 | 23.5 ± 1.9 | Stable antidepressants | No | Left DLPFC | 20 Hz | 90% | 800 (10) | 8,000 | 2 weeks |
|  | Sham (rotation 90°) | MDD | DSM-IV | Yes | 10/10 | 6/4 | 47.3 ± 3.5 | HAMD-17 | 24.9 ± 1.9 | Stable antidepressants | No |  |  |  |  |  |  |
| Cheng et al., 2022, China^[16]^ | Prolonged iTBS | MDD | DSM-IV | No | 26/26 | 9/17 | 46.7 ± 14.5 | HAMD-17 | 21.8 ± 3.1 | Drug-free | Yes | Left DLPFC | 50 Hz bursts at 5 Hz | 80% | 1,800 (10) | 18,000 | 2 weeks |
|  | rTMS | MDD | DSM-IV | No | 23/23 | 8/15 | 48.2 ± 14.1 | HAMD-17 | 23.4 ± 4.0 | Drug-free | Yes | Left DLPFC | 10 Hz | 100% | 1,600 (10) | 16,000 | 2 weeks |
|  | Matched sham coil | MDD | DSM-IV | No | 26/26 | 5/21 | 45.4 ± 12.4 | HAMD-17 | 23.4 ± 3.2 | Drug-free | Yes |  |  |  |  |  |  |
| Cheng et al., 2022, China^[17]^ | Standard iTBS | MDD | DSM-IV | Yes | 11/11 | 5/6 | 46.0 ± 15.0 | HAMD-17 | 24.0 ± 5.0 | Drug-free | Yes | DMPFC | 50 Hz bursts at 5 Hz | 80% | 600 (30) | 18,000 | 3 weeks |
|  | Prolonged iTBS | MDD | DSM-IV | Yes | 12/12 | 5/7 | 46.0 ± 18.0 | HAMD-17 | 25.0 ± 6.0 | Drug-free | Yes | DMPFC | 50 Hz bursts at 5 Hz | 80% | 1,800 (30) | 54,000 | 3 weeks |
|  | Matched sham coil | MDD | DSM-IV | Yes | 11/11 | 4/7 | 47.0 ± 16.0 | HAMD-17 | 24.0 ± 4.0 | Drug-free | Yes |  |  |  |  |  |  |
| Chistyakov et al., 2015, Israel^[18]^ | Continuous TBS | Mixed | DSM-IV | No | 15/15 | 5/10 | 52.7 ± 11.1 | HAMD-21 | 26.7 ± 3.9 | Stable Psychotropic drugs | No | Right DLPFC | 50 Hz bursts at 5 Hz | 100% | 3,600 (10) | 36,000 | 2 weeks |
|  | Matched sham coil | Mixed | DSM-IV | No | 14/13 | 6/8 | 50.9 ± 17.3 | HAMD-21 | 24.8 ± 3.2 | Stable Psychotropic drugs | No |  |  |  |  |  |  |
| Chou et al., 2020, China^[19]^ | TBS | MDD | DSM-IV | No | 30/27 | 12/15 | 43.6 ± 16.6 | HAMD-21 | 24.3 ± 3.9 | Benzodiazepine | Yes | Bilateral DLPFC | 50 Hz bursts at 5 Hz | 80% | 1,200 (10) | 12,000 | 3 weeks |
|  | Matched sham coil | MDD | DSM-IV | No | 30/26 | 11/15 | 42.3 ± 11.1 | HAMD-21 | 24.8 ± 5.3 | Benzodiazepine | Yes |  |  |  |  |  |  |
| Chou et al., 2023, China^[20]^ | TBS | MDD | DSM-IV | No | 17/17 | 8/9 | 49.2 ± 14.9 | HAMD-21 | 24.4 ± 3.0 | Stable antidepressants | No | Bilateral DLPFC | 50 Hz bursts at 5 Hz | 80% | 1,200 (10) | 12,000 | 2 weeks |
|  | Matched sham coil | MDD | DSM-IV | No | 11/11 | 5/6 | 43.5 ± 12.1 | HAMD-21 | 25.1 ± 6.9 | Stable antidepressants | No |  |  |  |  |  |  |
| Cole et al., 2022, USA^[21]^ | Accelerated iTBS | MDD | DSM-IV | Yes | 14/14 | 9/5 | 49.0 ± 15.0 | MADRS | 31.0 ± 4.0 | Stable antidepressants | No | Left DLPFC | 50 Hz bursts at 5 Hz | 90% | 1,800 (50) | 90,000 | 1 week |
|  | Matched sham coil | MDD | DSM-IV | Yes | 15/15 | 10/5 | 52.0 ± 16.0 | MADRS | 35.0 ± 6.0 | Stable antidepressants | No |  |  |  |  |  |  |
| Concerto et al., 2015, Italy^[22]^ | rTMS | MDD | DSM-IV | Yes | 15/15 | 9/6 | 51.0 ± 6.5 | HAMD-21 | 22.3 ± 2.2 | Stable Psychotropic drugs | No | Left DLPFC | 10 Hz | 120% | 3,000 (20) | 60,000 | 4 weeks |
|  | Sham (rotation 45°) | MDD | DSM-IV | Yes | 15/15 | 8/7 | 53.0 ± 6.7 | HAMD-21 | 21.0 ± 1.05 | Stable Psychotropic drugs | No |  |  |  |  |  |  |
| Dai et al., 2021, China^[23]^ | rTMS | MDD | ICD-10 | No | 55/40 | 13/27 | 44.5 ± 9.1 | HAMD-21 | 34.8 ± 5.4 | Escitalopram | No | Left DLPFC | 10 Hz | 100% | 8,000 (20) | 160,000 | 4 weeks |
|  | Sham (rotation 90°) | MDD | ICD-10 | No | 55/49 | 16/33 | 42.1 ± 9.5 | HAMD-21 | 33.9 ± 7.7 | Escitalopram | No |  |  |  |  |  |  |
| Dellink et al., 2024, Belgium^[24]^ | Continuous TBS | BD | DSM-IV | No | 18/18 | 5/13 | 48.0 ± 7.0 | HAMD-17 | 21.9 ± 3.9 | Stable Psychotropic drugs | No | Right DLPFC | 50 Hz bursts at 5 Hz | 110% | 900 (20) | 18,000 | 4 days |
|  | Matched sham coil | BD | DSM-IV | No | 19/19 | 6/13 | 51.0 ± 14.0 | HAMD-17 | 22.0 ± 3.0 | Stable Psychotropic drugs | No |  |  |  |  |  |  |
| Dunlop et al., 2020, Canada^[25]^ | rTMS | MDD | DSM-IV | Yes | 38/35 | 78/120 | 39.4 ± 11.6 | HAMD-17 | 22.5 ± 3.5 | Stable Psychotropic drugs | No | DMPFC | 20 Hz | 120% | 3040 (30) | 91,200 | 15 days |
|  | rTMS | MDD | DSM-IV | Yes | 41/37 |  |  | HAMD-17 | 22.0 ± 3.5 | Stable Psychotropic drugs | No | DMPFC | 1 Hz | 120% | 720 (30) | 21,600 | 15 days |
|  | Matched sham coil | MDD | DSM-IV | Yes | 41/36 |  |  | HAMD-17 | 22.3 ± 2.8 | Stable Psychotropic drugs | No |  |  |  |  |  |  |
| Duprat et al., 2016, Belgium^[26]^ | Accelerated iTBS | MDD | ICD-10 | Yes | 22/22 | 8/14 | 41.7 ± 11.8 | HAMD-17 | 21.1 ± 4.7 | Benzodiazepine | Yes | Left DLPFC | 50 Hz bursts at 5 Hz | 110% | 1,620 (20) | 32,400 | 4 days |
|  | Matched sham coil | MDD | ICD-10 | Yes | 25/25 | 10/15 |  | HAMD-17 | 21.5 ± 6.3 | Benzodiazepine | Yes |  |  |  |  |  |  |
| Eschweiler et al., 2000, Germany^[25, 27]^ | rTMS | MDD | DSM-IV | No | 5/5 | NA | 57.0 ± 8.0 | HAMD-21 | 27.4 ± 4.6 | Stable Psychotropic drugs | No | Left DLPFC | 10 Hz | 90% | 2,000 (5) | 10,000 | 1 week |
|  | Sham (rotation 90°) | MDD | DSM-IV | No | 5/5 | NA |  | HAMD-21 | 20.2 ± 3.8 | Stable Psychotropic drugs | No |  |  |  |  |  |  |
| Fitzgerald et al., 2003, Australia^[28]^ | rTMS | Mixed | DSM-IV | Yes | 20/20 | 12/8 | 42.2 ± 9.8 | MADRS | 36.1 ± 7.5 | Stable Psychotropic drugs | No | Left DLPFC | 10 Hz | 100% | 1,000 (10) | 10,000 | 2 weeks |
|  | rTMS | Mixed | DSM-IV | Yes | 20/20 | 13/7 | 45.6 ± 11.5 | MADRS | 37.7 ± 8.4 | Stable Psychotropic drugs | No | Right DLPFC | 1 Hz | 100% | 300 (10) | 3,000 | 2 weeks |
|  | Sham (rotation 45°) | Mixed | DSM-IV | Yes | 20/20 | 9/11 | 49.2 ± 14.2 | MADRS | 35.7 ± 8.1 | Stable Psychotropic drugs | No |  |  |  |  |  |  |
| Fitzgerald et al., 2006, Australia^[29]^ | rTMS | Mixed | DSM-IV | No | 25/25 | 10/15 | 46.8 ± 10.7 | MADRS | 34.0 ± 5.9 | Stable Psychotropic drugs | No | Bilateral DLPFC | Right 1 Hz; Left 10 Hz | Right: 100%, Left: 110% | Right: 420 (10), Left: 750 (10) | 11,700 | 2 weeks |
|  | Sham (rotation 45°) | Mixed | DSM-IV | No | 25/22 | 9/16 | 43.7 ± 10.2 | MADRS | 34.1 ± 5.2 | Stable Psychotropic drugs | No |  |  |  |  |  |  |
| Fitzgerald et al., 2012, Australia^[30]^ | rTMS | MDD | DSM-IV | Yes | 24/24 | 9/15 | 43.4 ± 12.7 | HAMD-17 | 23.7 ± 3.8 | Stable Psychotropic drugs | No | Left DLPFC | 10 Hz | 120% | 1,500 (15) | 22,500 | 3 weeks |
|  | rTMS | MDD | DSM-IV | Yes | 22/19 | 8/14 | 40.5 ± 15.5 | HAMD-17 | 24.3 ± 3.6 | Stable Psychotropic drugs | No | Bilateral DLPFC | Right 1 Hz; Left 10 Hz | 120% | Right: 900 (15), Left: 1,500 (15) | 36,000 | 3 weeks |
|  | Sham (rotation 45°) | MDD | DSM-IV | Yes | 20/17 | 12/8 | 44.9 ± 15.7 | HAMD-17 | 22.8 ± 2.1 | Stable Psychotropic drugs | No |  |  |  |  |  |  |
| Fitzgerald et al., 2016, Australia^[31]^ | rTMS | BD | ICD-10 | No | 23/23 | 10/13 | 43.6 ± 12.6 | HAMD-17 | 23.2 ± 4.0 | Stable Psychotropic drugs | No | Bilateral DLPFC | Right 1 Hz; Left 10 Hz | 110% | Right: 1,000 (20), Left: 1,000 (20) | 40,000 | 4 weeks |
|  | Sham (rotation 45°) | BD | ICD-10 | No | 23/23 | 10/13 | 49.7 ± 11.0 | HAMD-17 | 23.0 ± 5.1 | Stable Psychotropic drugs | No |  |  |  |  |  |  |
| Frick et al., 2021, Sweden^[32]^ | iTBS | BD | DSM-V | No | 19/19 | 9/10 | 30.1 ± 10.3 | MADRS-S | 30.2 ± 8.6 | Stable Psychotropic drugs | No | DMPFC | 50 Hz bursts at 5 Hz | 90% | 1,200 (20–30) | 24,000–36,000 | 10–15 days |
|  | Matched sham coil | BD | DSM-V | No | 21/21 | 10/11 | 28.7 ± 8.8 | MADRS-S | 30.2 ± 8.0 | Stable Psychotropic drugs | No |  |  |  |  |  |  |
| García-Toro et al., 2001, Spain^[33]^ | rTMS | MDD | DSM-IV | No | 11/11 | 5/6 | 43.2 ± 13.1 | HAMD-21 | 25.9 ± 6.4 | Sertraline, benzodiazepine | Yes | Left DLPFC | 20 Hz | 90% | 1,200 (10) | 12,000 | 2 weeks |
|  | Sham (rotation 90°) | MDD | DSM-IV | No | 11/11 | 5/6 | 45.0 ± 18.3 | HAMD-21 | 26.6 ± 6.4 | Sertraline, benzodiazepine | Yes |  |  |  |  |  |  |
| García-Toro et al., 2001, Spain^[34]^ | rTMS | MDD | DSM-IV | Yes | 17/17 | 10/7 | 51.5 ± 15.9 | HAMD-21 | 27.1 ± 6.7 | Stable Psychotropic drugs | No | Left DLPFC | 20 Hz | 90% | 1,800 (10) | 18,000 | 2 weeks |
|  | Sham (rotation 90°) | MDD | DSM-IV | Yes | 18/18 | 10/8 | 50.0 ± 11.0 | HAMD-21 | 25.6 ± 4.9 | Stable Psychotropic drugs | No |  |  |  |  |  |  |
| Garcia-Toro et al., 2006, Spain^[35]^ | rTMS | MDD | DSM-IV | Yes | 10/10 | 6/4 | 48.5 ± 13.3 | HAMD-21 | 27.3 ± 5.0 | Stable Psychotropic drugs | No | Bilateral DLPFC | Right 1 Hz; Left 20 Hz | 110% | Right: 1,500 (10), Left: 1,500 (10) | 30,000 | 2 weeks |
|  | Sham (rotation 45°) | MDD | DSM-IV | Yes | 10/10 | 3/7 | 47.2 ± 11.8 | HAMD-21 | 25.1 ± 7.3 | Stable Psychotropic drugs | No |  |  |  |  |  |  |
| George et al., 1997, USA^[36]^ | rTMS | Mixed | DSM-IV | No | 6/6 | 4/2 | 42.4 ± 15.5 | HAMD-21 | 30.0 ± 4.0 | Drug-free | Yes | Left DLPFC | 20 Hz | 80% | 800 (10) | 8,000 | 2 weeks |
|  | Matched sham coil | Mixed | DSM-IV | No | 6/6 | 4/2 | 41.0 ± 8.3 | HAMD-21 | 26.0 ± 3.0 | Drug-free | Yes |  |  |  |  |  |  |
| George et al., 2000, USA^[37]^ | rTMS | Mixed | DSM-IV | No | 10/10 | 4/6 | 43.0 ± 11.0 | HAMD-21 | 28.1 ± 6.0 | Benzodiazepine, mood stabilizers | Yes | Left DLPFC | 5 Hz | 100% | 1,600 (10) | 16,000 | 2 weeks |
|  | rTMS | Mixed | DSM-IV | No | 10/10 | 3/7 | 41.4 ± 11.0 | HAMD-21 | 26.3 ± 5.9 | Benzodiazepine, mood stabilizers | Yes | Left DLPFC | 20 Hz | 100% | 1,600 (10) | 16,000 | 2 weeks |
|  | Sham (rotation 45°) | Mixed | DSM-IV | No | 10/10 | 4/6 | 48.5 ± 8.0 | HAMD-21 | 23.8 ± 4.1 | Benzodiazepine, mood stabilizers | Yes |  |  |  |  |  |  |
| George et al., 2010, USA^[38]^ | rTMS | MDD | DSM-IV | Yes | 92/83 | 34/58 | 47.7 ± 10.6 | HAMD-24 | 26.3 ± 5.0 | Benzodiazepine, zolpidem | Yes | Left DLPFC | 10 Hz | 120% | 3,000 (15–30) | 45,000–90,000 | 3–6 weeks |
|  | Matched sham coil | MDD | DSM-IV | Yes | 98/91 | 48/50 | 46.5 ± 12.3 | HAMD-24 | 26.5 ± 4.8 | Benzodiazepine, zolpidem | Yes |  |  |  |  |  |  |
| Hernández-Ribas et al., 2012, Spain^[39]^ | rTMS | MDD | DSM-IV | No | 10/10 | 2/8 | 42.6 ± 5.6 | HAMD-17 | 19.7 ± 3.8 | Stable Psychotropic drugs | No | Left DLPFC | 15 Hz | 100% | 1,500 (15) | 22,500 | 3 weeks |
|  | Matched sham coil | MDD | DSM-IV | No | 11/11 | 3/8 | 50.1 ± 8.1 | HAMD-17 | 16.6 ± 2.4 | Stable Psychotropic drugs | No |  |  |  |  |  |  |
| Herwig et al., 2003, Germany^[40]^ | rTMS | MDD | 1CD-10, DSM-IV | No | 13/13 | 5/8 | 41.6 ± 10.5 | HAMD-21 | NA | Stable Psychotropic drugs | No | DLPFC | 15 Hz | 110% | 3,000 (10) | 30,000 | 2 weeks |
|  | Matched sham coil | MDD | ICD-10, DSM-IV | No | 12/12 | 7/5 | 47.8 ± 10.0 | HAMD-21 | NA | Stable Psychotropic drugs | No |  |  |  |  |  |  |
| Herwig et al., 2007, Germany^[41]^ | rTMS | Mixed | ICD-10, DSM-IV | No | 62/52 | 18/44 | 50.0 ± 15.0 | HAMD-21 | 24.7 ± 5.4 | Venlafaxine / Mirtazapine monotherapy | No | Left DLPFC | 10 Hz | 110% | 2,000 (15) | 30,000 | 3 weeks |
|  | Sham (rotation 45°) | Mixed | ICD-10, DSM-IV | No | 65/53 | 33/32 | 49.0 ± 13.0 | HAMD-21 | 22.8 ± 4.8 | Venlafaxine / Mirtazapine monotherapy | No |  |  |  |  |  |  |
| Holtzheimer et al., 2004, USA^[42]^ | rTMS | MDD | DSM-IV | Yes | 7/7 | 3/4 | 40.4 ± 8.5 | HAMD-17 | 22.7 ± 5.3 | Drug-free | Yes | Left DLPFC | 10 Hz | 110% | 1,600 (10) | 16,000 | 2 weeks |
|  | Sham (rotation 45°) | MDD | DSM-IV | Yes | 8/8 | 5/3 | 45.4 ± 4.9 | HAMD-17 | 20.8 ± 6.3 | Drug-free | Yes |  |  |  |  |  |  |
| Höppner et al., 2003, Germany^[43]^ | rTMS | MDD | DSM-IV | No | 10/9 | 1/8 | 57.2 ± 12.2 | HAMD-21 | 21.5 ± 4.0 | Stable Psychotropic drugs | No | Left DLPFC | 20 Hz | 90% | 800 (10) | 8,000 | 2 weeks |
|  | rTMS | MDD | DSM-IV | No | 10/10 | 3/7 | 56.0 ± 10.7 | HAMD-21 | 22.0 ± 5.0 | Stable Psychotropic drugs | No | Right DLPFC | 1 Hz | 110% | 120 (10) | 1,200 | 2 weeks |
|  | Sham (rotation 90°) | MDD | DSM-IV | No | 10/10 | 4/6 | 56.0 ± 10.5 | HAMD-21 | 24.5 ± 5.5 | Stable Psychotropic drugs | No |  |  |  |  |  |  |
| Hu et al., 2016, China^[44]^ | rTMS | BD | DSM-IV | No | 11/11 | 5/6 | 27.4 ± 14.3 | HAMD-17 | NA | Quetiapine | Yes | Left DLPFC | 10 Hz | 80% | 1,200 (20) | 24,000 | 4 weeks |
|  | rTMS | BD | DSM-IV | No | 12/11 | 7/5 | 28.3 ± 10.3 | HAMD-17 | NA | Quetiapine | Yes | Right DLPFC | 1 Hz | 80% | 1,200 (20) | 24,000 | 4 weeks |
|  | Matched sham coil | BD | DSM-IV | No | 13/12 | 5/7 | 23.7 ± 9.8 | HAMD-17 | NA | Quetiapine | Yes |  |  |  |  |  |  |
| Huang et al., 2012, China^[45]^ | rTMS | MDD | DSM-IV | No | 30/28 | 9/19 | 32.8 ± 7.3 | HAMD-17 | 22.7 ± 2.3 | Citalopram | No | Left DLPFC | 10 Hz | 90% | 800 (10) | 8,000 | 2 weeks |
|  | Sham (rotation 90°) | MDD | DSM-IV | No | 30/28 | 8/20 | 31.4 ± 7.4 | HAMD-17 | 22.5 ± 2.7 | Citalopram | No |  |  |  |  |  |  |
| Januel et al., 2006, France^[46]^ | rTMS | MDD | DSM-IV | No | 11/11 | 2/9 | 38.6 ± 11.2 | HAMD-17 | 21.7 ± 3.5 | Oxazepam/zopiclone | Yes | Right DLPFC | 1 Hz | 90% | 120 (16) | 1,920 | 4 weeks |
|  | Matched sham coil | MDD | DSM-IV | No | 16/16 | 4/12 | 37.2 ± 11.7 | HAMD-17 | 22.5 ± 2.7 | Oxazepam/zopiclone | Yes |  |  |  |  |  |  |
| Kauffmann et al., 2004, USA^[47]^ | rTMS | MDD | DSM-IV | Yes | 7/7 | 1/11 | 51.7 ± 17.2 | HAMD-21 | 21.9 ± 2.3 | Stable Psychotropic drugs | No | Right PFC | 1 Hz | 110% | 120 (10) | 1,200 | 2 weeks |
|  | Sham (rotation 45°) | MDD | DSM-IV | Yes | 5/5 |  |  | HAMD-21 | 18.2 ± 2.2 | Stable Psychotropic drugs | No |  |  |  |  |  |  |
| Kazemi et al., 2022, Iran^[48]^ | rTMS | MDD | DSM-V | Yes | 25/25 | 10/15 | 32.0 ± 12.0 | HAMD-17 | 28.3 ± 7.4 | Stable antidepressants | No | Left DLPFC | 10 Hz | 100% | 3,750 (20) | 75,000 | 4 weeks |
|  | rTMS | MDD | DSM-V | Yes | 25/25 | 9/16 | 34.0 ± 11.0 | HAMD-17 | 32.8 ± 8.1 | Stable antidepressants | No | Bilateral DLPFC | Right 1 Hz; Left 10 Hz | 110% | 5,250 (20) | 105,000 | 4 weeks |
|  | Sham (rotation 90°) | MDD | DSM-V | Yes | 11/11 | 6/5 | 38.0 ± 11.0 | HAMD-17 | 26.2 ± 6.8 | Stable antidepressants | No |  |  |  |  |  |  |
| Koerselman et al., 2004, Netherlands^[49]^ | rTMS | MDD | DSM-IV | No | 26/26 | 14/12 | 51.0 ± 15.4 | HAMD-17 | 25.9 ± 4.3 | Stable Psychotropic drugs | No | Left DLPFC | 20 Hz | 80% | 800 (10) | 8,000 | 2 weeks |
|  | Sham (rotation 45°) | MDD | DSM-IV | No | 26/26 | 9/17 | 52.0 ± 13.2 | HAMD-17 | 25.9 ± 5.6 | Stable Psychotropic drugs | No |  |  |  |  |  |  |
| Kreuzer et al., 2015, Germany^[50]^ | rTMS | Mixed | DSM-IV | No | 15/15 | 7/8 | 46.1 ± 9.5 | HAMD-21 | 22.3 ± 4.7 | Stable Psychotropic drugs | No | Left DLPFC | 10 Hz | 110% | 2,000 (15) | 30,000 | 3 weeks |
|  | Matched sham coil | Mixed | DSM-IV | No | 12/12 | 4/8 | 43.8 ± 10.5 | HAMD-21 | 23.2 ± 4.7 | Stable Psychotropic drugs | No |  |  |  |  |  |  |
| Krstić et al., 2014, Serbia^[51]^ | rTMS | MDD | DSM-V | Yes | 11/11 | 0/11 | 50.7 ± 7.3 | HAMD-24 | 30.1 ± 3.5 | Stable Psychotropic drugs | No | Right DLPFC | 1 Hz | 110% | 300 (10) | 3,000 | 2 weeks |
|  | Matched sham coil | MDD | DSM-V | Yes | 8/8 | 0/8 | 46.1 ± 8.5 | HAMD-24 | 28.0 ± 2.7 | Stable Psychotropic drugs | No |  |  |  |  |  |  |
| Levkovitz et al., 2015, Israel, USA, Germany, Canada^[52]^ | Deep TMS | MDD | DSM-IV | Yes | 111/89 | 47/42 | 45.1 ± 11.7 | HAMD-21 | 23.5 ± 4.3 | Benzodiazepine | Yes | Left PFC | 18 Hz | 120% | 1,980 (20) | 39,600 | 4 weeks |
|  | Matched sham coil | MDD | DSM-IV | Yes | 122/92 | 48/44 | 47.6 ± 11.6 | HAMD-21 | 23.4 ± 3.7 | Benzodiazepine | Yes |  |  |  |  |  |  |
| Li et al., 2014, China^[53]^ | Continuous TBS | MDD | DSM-IV | Yes | 15/15 | 5/10 | 49.2 | HAMD-17 | 24.3 ± 5.5 | Stable Psychotropic drugs | No | Left DLPFC | 50 Hz bursts at 5 Hz | 80% | 1,800 (10) | 18,000 | 2 weeks |
|  | iTBS | MDD | DSM-IV | Yes | 15/15 | 7/8 | 42.4 | HAMD-17 | 23.1 ± 3.9 | Stable Psychotropic drugs | No | Left DLPFC | 50 Hz bursts at 5 Hz | 80% | 1,800 (10) | 18,000 | 2 weeks |
|  | iTBS + cTBS | MDD | DSM-IV | Yes | 15/15 | 4/11 | 42.5 | HAMD-17 | 25.4 ± 5.1 | Stable Psychotropic drugs | No | Bilateral DLPFC | 50 Hz bursts at 5 Hz | 80% | 3,600 (10) | 36,000 | 2 weeks |
|  | Sham (rotation 90°) | MDD | DSM-IV | Yes | 15/15 | 4/11 | 46.9 | HAMD-17 | 23.8 ± 3.2 | Stable Psychotropic drugs | No |  |  |  |  |  |  |
| Li et al., 2021, China^[54]^ | rTMS | MDD | DSM-IV | Yes | 35/35 | 24/11 | 47.1 ± 13.8 | HAMD-17 | 22.9 ± 3.8 | Sleep aid medication | Yes | Left DLPFC | 10 Hz | 100% | 3,000 (10) | 30,000 | 2 weeks |
|  | Prolonged iTBS | MDD | DSM-IV | Yes | 35/35 | 23/12 | 47.1 ± 14.2 | HAMD-17 | 22.5 ± 3.5 | Sleep aid medication | Yes | Left DLPFC | 50 Hz bursts at 5 Hz | 80% | 1,800 (10) | 18,000 | 2 weeks |
|  | Matched sham coil | MDD | DSM-IV | Yes | 35/35 | 24/11 | 47.1 ± 12.4 | HAMD-17 | 23.1 ± 3.5 | Sleep aid medication | Yes |  |  |  |  |  |  |
| Li et al., 2023, China^[55]^ | rTMS | MDD | DSM-V | No | 35/35 | 15/20 | 40.1 ± 12.9 | HAMD-24 | 23.0 ± 5.0 | Drug-free | Yes | Left DLPFC | 10 Hz | 110% | 1,600 (20) | 32,000 | 4 weeks |
|  | Matched sham coil | MDD | DSM-V | No | 31/31 | 17/14 | 38.1 ± 11.3 | HAMD-24 | 24.4 ± 6.7 | Drug-free | Yes |  |  |  |  |  |  |
| Li et al., 2023, China^[56]^ | rTMS | MDD | DSM-IV | No | 24/24 | 8/16 | 37.8 ± 2.7 | HAMD-17 | 25.6 ± 1.3 | Drug-free | Yes | Left DLPFC | 10 Hz | 120% | 6,000 (20) | 120,000 | 2 weeks |
|  | Prolonged iTBS | MDD | DSM-IV | No | 24/24 | 8/16 | 37.0 ± 3.1 | HAMD-17 | 24.5 ± 1.0 | Drug-free | Yes | Left DLPFC | 50 Hz bursts at 5 Hz | 80% | 3,600 (20) | 72,000 | 2 weeks |
|  | Matched sham coil | MDD | DSM-IV | No | 24/24 | 9/15 | 37.0 ± 2.7 | HAMD-17 | 24.1 ± 1.2 | Drug-free | Yes |  |  |  |  |  |  |
| Li et al., 2024, China^[57]^ | rTMS | MDD | DSM-IV | No | 60/53 | 24/36 | 30.4 ± 12.3 | HAMD-24 | 26.3 ± 4.6 | Stable Psychotropic drugs | No | Left DLPFC | 10 Hz | 100% | 1,600 (10) | 16,000 | 2 weeks |
|  | Matched sham coil | MDD | DSM-IV | No | 60/52 | 23/37 | 32.0 ± 15.4 | HAMD-24 | 26.1 ± 5.5 | Stable Psychotropic drugs | No |  |  |  |  |  |  |
| Lingeswaran, 2011, India^[58]^ | rTMS | MDD | DSM-IV | No | 9/9 | 3/6 | 34.0 ± 10.5 | HAMD-17 | 22.8 ± 3.7 | Stable Psychotropic drugs | No | Left DLPFC | 10 Hz | 100% | 500 (12) | 6,000 | 2 weeks |
|  | Sham (rotation 90°) | MDD | DSM-IV | No | 14/14 | 6/8 | 37.2 ± 11.8 | HAMD-17 | 22.0 ± 3.1 | Stable Psychotropic drugs | No |  |  |  |  |  |  |
| Loo et al., 2003, Australia^[59]^ | rTMS | MDD | DSM-IV | Yes | 9/9 | 3/6 | 54.9 ± 18.0 | HAMD | 25.0 ± 3.5 | Stable Psychotropic drugs | No | Bilateral DLPFC | 15 Hz | 90% | 1,800 (15) | 27,000 | 3 weeks |
|  | Matched sham coil | MDD | DSM-IV | Yes | 10/10 | 4/6 | 48.4 ± 10.9 | HAMD | 26.0 ± 3.5 | Stable Psychotropic drugs | No |  |  |  |  |  |  |
| Loo et al., 2007, Australia^[60]^ | rTMS | MDD | DSM-IV | Yes | 19/18 | 9/10 | 49.8 ± 2.5 | HAMD-17 | 19.2 ± 3.7 | Stable Psychotropic drugs | No | Left DLPFC | 10 Hz | 110% | 1,500 (20) | 30,000 | 2 weeks |
|  | Matched sham coil | MDD | DSM-IV | Yes | 19/18 | 11/8 | 45.7 ± 15.0 | HAMD-17 | 20.9 ± 4.2 | Stable Psychotropic drugs | No |  |  |  |  |  |  |
| Mak et al., 2021, China^[61]^ | rTMS | BD | DSM-V | No | 26/23 | 8/18 | 40.7 ± 11.4 | MADRS | 27.5 ± 5.2 | Stable Psychotropic drugs | No | Right DLPFC | 1 Hz | 110% | 300 (15) | 4,500 | 3 weeks |
|  | Matched sham coil | BD | DSM-V | No | 28/26 | 10/18 | 39.4 ± 11.3 | MADRS | 27.0 ± 5.1 | Stable Psychotropic drugs | No |  |  |  |  |  |  |
| Mallik et al., 2022, India^[62]^ | Continuous TBS | BD | ICD-10 | No | 11/11 | 5/6 | 43.8 ± 13.0 | HAMD-17 | 27.6 ± 9.1 | Stable Psychotropic drugs | No | Right DLPFC | 50 Hz bursts at 5 Hz | 80% | 600 (15) | 9,000 | 1 week |
|  | Matched sham coil | BD | ICD-10 | No | 8/8 | 5/3 | 43.6 ± 10.6 | HAMD-17 | 26.3 ± 9.2 | Stable Psychotropic drugs | No |  |  |  |  |  |  |
| Martinot et al., 2010, France^[63]^ | rTMS | Mixed | DSM-IV | Yes | 19/18 | NA | 48.2 ± 7.8 | HAMD-21 | 26.0 ± 6.4 | Stable Psychotropic drugs | No | Left DLPFC | 10 Hz | 90% | 1,600 (10) | 16,000 | 2 weeks |
|  | Sham (rotation 90°) | Mixed | DSM-IV | Yes | 14/14 | NA | 46.6 ± 10.3 | HAMD-21 | 25.9 ± 6.7 | Stable Psychotropic drugs | No |  |  |  |  |  |  |
| Matsuda et al., 2020, Japan^[64]^ | Deep TMS | Mixed | DSM-IV | Yes | 20/18 | 18/2 | 43.4 ± 5.5 | HAMD-21 | 19.4 ± 8.2 | Stable Psychotropic drugs | No | Left PFC | 18 Hz | 120% | 1,980 (20–30) | 39,600–59,400 | 4–6 weeks |
|  | Matched sham coil | Mixed | DSM-IV | Yes | 20/20 | 19/1 | 45.2 ± 7.0 | HAMD-21 | 20.5 ± 4.1 | Stable Psychotropic drugs | No |  |  |  |  |  |  |
| McDonald et al., 2006, USA^[65]^ | rTMS | Mixed | DSM-IV | Yes | 50/50 | 23/27 | 49.0 | HAMD-17 | 27.0 ± 3.2 | Stable Psychotropic drugs | No | Bilateral DLPFC | Right 1 Hz; Left 10 Hz | 110% | 1,600 (10) | 16,000 | 2 weeks |
|  | Sham (rotation 90°) | Mixed | DSM-IV | Yes | 12/12 | 7/5 | 54.0 | HAMD-17 | 27.0 ± 2.0 | Stable Psychotropic drugs | No |  |  |  |  |  |  |
| McGirr et al., 2021, Canada^[66]^ | iTBS | BD | DSM-V | No | 18/15 | 7/11 | 44.8 ± 13.7 | MADRS | 32.3 ± 4.0 | Stable Psychotropic drugs | No | Left DLPFC | 50 Hz bursts at 5 Hz | 120% | 600 (20) | 12,000 | 4 weeks |
|  | Matched sham coil | BD | DSM-V | No | 19/16 | 7/12 | 43.0 ± 14.3 | MADRS | 31.5 ± 5.2 | Stable Psychotropic drugs | No |  |  |  |  |  |  |
| Mogg et al., 2008, UK^[67]^ | rTMS | BD | DSM-IV | Yes | 29/28 | 13/16 | 55.0 ± 18.0 | HAMD-17 | 20.5 ± 4.4 | Stable Psychotropic drugs | No | Left DLPFC | 10 Hz | 110% | 1,000 (10) | 10,000 | 2 weeks |
|  | Matched sham coil | BD | DSM-IV | Yes | 30/29 | 9/21 | 52.0 ± 15.5 | HAMD-17 | 21.6 ± 4.7 | Stable Psychotropic drugs | No |  |  |  |  |  |  |
| Murgaš et al., 2023, Slovakia^[68]^ | TBS | MDD | DSM-IV | Yes | 8/8 | 3/5 | 35.9 ± 8.4 | HAMD-17 | 18.5 ± 3.6 | Stable Psychotropic drugs | No | Bilateral DLPFC | 50 Hz bursts at 5 Hz | 120% | 1,200 (15) | 18,000 | 3 weeks |
|  | Sham (rotation 90°) | MDD | DSM-IV | Yes | 3/3 | 0/3 | 40.3 ± 6.0 | HAMD-17 | 22.0 ± 2.7 | Stable Psychotropic drugs | No |  |  |  |  |  |  |
| Nahas et al., 2003, USA^[69]^ | rTMS | BD | DSM-IV | No | 11/11 | 4/7 | 42.4 ± 7.3 | HAMD-28 | 32.5 ± 4.3 | Stable Psychotropic drugs | No | Left DLPFC | 5 Hz | 110% | 1,600 (10) | 16,000 | 2 weeks |
|  | Sham (rotation 45°) | BD | DSM-IV | No | 12/12 | 5/7 | 43.4 ± 9.3 | HAMD-28 | 32.8 ± 7.6 | Stable Psychotropic drugs | No |  |  |  |  |  |  |
| Novák et al., 2024, Czech Republic^[70]^ | rTMS | BD | DSM-IV | No | 20/15 | 8/12 | 48.9 ± 12.8 | MADRS | 26.1 ± 4.2 | Stable Psychotropic drugs | No | Left DLPFC | 10 Hz | 100% | 1,200 (20) | 24,000 | 4 weeks |
|  | Matched sham coil | BD | DSM-IV | No | 20/15 | 8/12 | 43.9 ± 10.7 | MADRS | 26.9 ± 4.3 | Stable Psychotropic drugs | No |  |  |  |  |  |  |
| O'Reardon et al., 2007, USA^[71]^ | rTMS | MDD | DSM-IV | No | 155/143 | 69/86 | 47.9 ± 11.0 | HAMD-17 | 22.6 ± 3.3 | Lorazepam | Yes | Left DLPFC | 10 Hz | 120% | 3,000 (20–30) | 60,000–90,000 | 4–6 weeks |
|  | Matched sham coil | MDD | DSM-IV | No | 146/134 | 72/74 | 48.7 ± 10.6 | HAMD-17 | 22.9 ± 3.5 | Lorazepam | Yes |  |  |  |  |  |  |
| Ortega Rumi et al., 2005, Brazil^[72]^ | rTMS | MDD | DSM-IV | No | 22/22 | 3/19 | 39.3 ± 12.8 | HAMD-17 | 29.7 ± 6.4 | Amitriptyline | Yes | Left DLPFC | 5 Hz | 120% | 1,250 (20) | 25,000 | 4 weeks |
|  | Matched sham coil | MDD | DSM-IV | No | 24/24 | 4/20 | 38.9 ± 8.8 | HAMD-17 | 30.9 ± 5.5 | Amitriptyline | Yes |  |  |  |  |  |  |
| Padberg et al., 2002, Germany^[73]^ | rTMS | MDD | DSM-IV | Yes | 10/10 | 4/6 | 62.1 ± 4.6 | HAMD-21 | 22.9 ± 3.5 | Stable Psychotropic drugs | No | Left DLPFC | 10 Hz | 100% | 1,500 (10) | 15,000 | 2 weeks |
|  | rTMS | MDD | DSM-IV | Yes | 10/10 | 3/7 | 60.3 ± 4.1 | HAMD-21 | 23.6 ± 1.9 | Stable Psychotropic drugs | No | Left DLPFC | 10 Hz | 90% | 1,500 (10) | 15,000 | 2 weeks |
|  | Sham (rotation 90°) | MDD | DSM-IV | Yes | 10/10 | 2/8 | 52.7 ± 5.7 | HAMD-21 | 21.9 ± 1.8 | Stable Psychotropic drugs | No |  |  |  |  |  |  |
| Pallanti et al., 2010, Italy^[74]^ | rTMS | MDD | DSM-IV | Yes | 20/20 | 8/12 | 51.2 ± 12.5 | HAMD-17 | 28.0 ± 6.0 | Stable Psychotropic drugs | No | Right DLPFC | 1 Hz | 110% | 420 (15) | 6,000 | 3 weeks |
|  | rTMS | MDD | DSM-IV | Yes | 20/20 | 9/11 | 47.6 ± 12.3 | HAMD-17 | 28.8 ± 6.0 | Stable Psychotropic drugs | No | Bilateral DLPFC | Right 1 Hz; Left 10 Hz | 100% | 1,420 (15) | 21,300 | 3 weeks |
|  | Matched sham coil | MDD | DSM-IV | Yes | 20/20 | 8/12 | 47.9 ± 9.1 | HAMD-17 | 29.1 ± 3.5 | Stable Psychotropic drugs | No |  |  |  |  |  |  |
| Pan et al., 2020, China^[75]^ | rTMS | MDD | DSM-IV | No | 21/21 | 2/19 | 18.1 ± 3.9 | HAMD-24 | 38.3 ± 7.9 | Escitalopram | No | Left DLPFC | 10 Hz | 100% | 6,000 (7) | 42,000 | 1 week |
|  | Sham (rotation 90°) | MDD | DSM-IV | No | 21/21 | 5/16 | 21.4 ± 6.8 | HAMD-24 | 35.8 ± 8.9 | Escitalopram | No |  |  |  |  |  |  |
| Pan et al., 2023, China^[76]^ | rTMS | MDD | DSM-IV | No | 31/31 | 10/21 | 18.0 ± 4.0 | HAMD-24 | 37.3 ± 9.7 | Escitalopram | No | Left DLPFC | 10 Hz | 100% | 6,000 (7) | 42,000 | 1 week |
|  | Sham (rotation 90°) | MDD | DSM-IV | No | 28/28 | 8/20 | 20.6 ± 5.8 | HAMD-24 | 35.4 ± 8.9 | Escitalopram | No |  |  |  |  |  |  |
| Plewnia et al., 2014, Germany^[77]^ | TBS | MDD | DSM-IV | No | 16/16 | 8/8 | 46.9 ± 13.2 | HAMD | 23.6 ± 5.3 | Stable Psychotropic drugs | No | Bilateral DLPFC | 50 Hz bursts at 5 Hz | 80% | 1,200 (30) | 36,000 | 6 weeks |
|  | Sham (rotation 45°) | MDD | DSM-IV | No | 16/16 | 4/12 | 49.0 ± 13.6 | HAMD | 22.2 ± 5.9 | Stable Psychotropic drugs | No |  |  |  |  |  |  |
| Prasser et al., 2014, Germany^[78]^ | rTMS | MDD | DSM-IV | No | 17/17 | 8/9 | 50.4 ± 9.9 | HAMD-21 | 25.0 ± 4.4 | Stable Psychotropic drugs | No | Bilateral DLPFC | Right 1 Hz; Left 10 Hz | 110% | 2,000 (15) | 30,000 | 3 weeks |
|  | TBS | MDD | DSM-IV | No | 20/20 | 10/10 | 48.2 ± 10.9 | HAMD-21 | 27.4 ± 6.5 | Stable Psychotropic drugs | No | Bilateral DLPFC | 50 Hz bursts at 5 Hz | 80% | 2,400 (15) | 36,000 | 3 weeks |
|  | Matched sham coil | MDD | DSM-IV | No | 17/17 | 9/8 | 42.6 ± 12.4 | HAMD-21 | 25.3 ± 5.4 | Stable Psychotropic drugs | No |  |  |  |  |  |  |
| Pu et al., 2022, China^[79]^ | rTMS | MDD | ICD-10 | No | 50/42 | 19/31 | 38.5 ± 10.1 | HAMD-17 | 22.8 ± 6.4 | Agomelatine | No | Left DLPFC | 10 Hz | 120% | 2,000 (20) | 40,000 | 4 weeks |
|  | Sham (rotation 90°) | MDD | ICD-10 | No | 50/40 | 22/28 | 36.7 ± 9.6 | HAMD-17 | 21.5 ± 6.6 | Agomelatine | No |  |  |  |  |  |  |
| Ramos et al., 2025, Brazil^[80]^ | Accelerated iTBS | MDD | DSM-V | Yes | 50/45 | 8/42 | 44.5 ± 8.2 | HAMD-17 | 20.6 ± 2.3 | Stable Psychotropic drugs | No | Left DLPFC | 50 Hz bursts at 5 Hz | 100% | 1,200 (45) | 54,000 | 3 weeks |
|  | Matched sham coil | MDD | DSM-V | Yes | 50/44 | 8/42 | 40.6 ± 9.0 | HAMD-17 | 20.5 ± 2.3 | Stable Psychotropic drugs | No |  |  |  |  |  |  |
| Ray et al., 2011, India^[81]^ | rTMS | MDD | ICD-10 | No | 21/20 | 5/15 | 36.8 ± 12.3 | HAMD | 29.6 ± 3.8 | Stable Psychotropic drugs | No | Left DLPFC | 10 Hz | 90% | 1,200 (10) | 12,000 | 2 weeks |
|  | Sham (rotation 45°) | MDD | ICD-10 | No | 20/20 | 3/17 | 31.3 ± 9.3 | HAMD | 29.4 ± 5.7 | Stable Psychotropic drugs | No |  |  |  |  |  |  |
| Rossini et al., 2005, Italy^[82]^ | rTMS | MDD | DSM-IV | Yes | 18/18 | 6/12 | 57.4 ± 8.7 | HAMD-21 | 28.8 ± 3.1 | Stable Psychotropic drugs | No | Left DLPFC | 15 Hz | 100% | 600 (10) | 6,000 | 2 weeks |
|  | rTMS | MDD | DSM-IV | Yes | 19/18 | 4/15 | 54.0 ± 11.2 | HAMD-21 | 28.6 ± 2.7 | Stable Psychotropic drugs | No | Left DLPFC | 15 Hz | 80% | 600 (10) | 6,000 | 2 weeks |
|  | Sham (rotation 90°) | MDD | DSM-IV | Yes | 17/16 | 6/11 | 56.3 ± 12.6 | HAMD-21 | 28.7 ± 2.1 | Stable Psychotropic drugs | No |  |  |  |  |  |  |
| Rossini et al., 2005, Italy^[83]^ | rTMS | MDD | DSM-IV | No | 50/49 | 11/39 | 48.4 ± 13.7 | HAMD-21 | 25.1 ± 3.5 | Stable SSRI/SNRI | No | Left DLPFC | 15 Hz | 100% | 900 (10) | 9,000 | 2 weeks |
|  | Sham (rotation 90°) | MDD | DSM-IV | No | 49/47 | 9/40 | 46.4 ± 12.1 | HAMD-21 | 25.1 ± 3.1 | Stable SSRI/SNRI | No |  |  |  |  |  |  |
| Rothärmel et al., 2021, France^[84]^ | rTMS | Mixed | DSM-IV | Yes | 28/26 | 11/17 | 48.4 ± 13.0 | HAMD-21 | 26.2 ± 5.5 | Stable Psychotropic drugs | No | Left DLPFC | 20 Hz | 90% | 800 (5) | 4,000 | 1 week |
|  | Matched sham coil | Mixed | DSM-IV | Yes | 28/26 | 12/16 | 51.6 ± 15.4 | HAMD-21 | 28.3 ± 4.8 | Stable Psychotropic drugs | No |  |  |  |  |  |  |
| Sheline et al., 2024, USA^[85]^ | Accelerated iTBS | BD | DSM-IV | Yes | 12/12 | 6/6 | 43.1 ± 15.2 | MADRS | 30.4 ± 4.8 | Stable Psychotropic drugs | No | Left DLPFC | 50 Hz bursts at 5 Hz | 90% | 1,800 (50) | 90,000 | 10 days |
|  | Matched sham coil | BD | DSM-IV | Yes | 12/12 | 6/6 | 43.6 ± 19.2 | MADRS | 28.0 ± 5.4 | Stable Psychotropic drugs | No |  |  |  |  |  |  |
| Speer et al., 2014, USA^[86]^ | rTMS | Mixed | DSM-IV | No | 16/16 | 6/10 | 40.4 ± 11.7 | HAMD-28 | 32.2 ± 9.7 | Drug-free | Yes | Left DLPFC | 1 Hz or 20 Hz | 110% | 1,600 (15) | 24,000 | 3 weeks |
|  | Sham (rotation 45°) | Mixed | DSM-IV | No | 8/8 | 5/3 | 44.9 ± 9.1 | HAMD-28 | 24.0 ± 4.6 | Drug-free | Yes |  |  |  |  |  |  |
| Stern et al., 2007, USA^[87]^ | rTMS | MDD | DSM-IV | Yes | 10/10 | 4/6 | 53.2 ± 12.0 | HAMD-21 | 27.8 ± 3.2 | Drug-free | Yes | Left DLPFC | 10 Hz | 110% | 1,600 (10) | 16,000 | 2 weeks |
|  | rTMS | MDD | DSM-IV | Yes | 10/8 | 4/6 | 52.4 ± 9.4 | HAMD-21 | 27.6 ± 3.9 | Drug-free | Yes | Left DLPFC | 1 Hz | 110% | 1,600 (10) | 16,000 | 2 weeks |
|  | rTMS | MDD | DSM-IV | Yes | 10/10 | 3/7 | 52.8 ± 9.5 | HAMD-21 | 27.9 ± 3.8 | Drug-free | Yes | Right DLPFC | 1 Hz | 110% | 1,600 (10) | 16,000 | 2 weeks |
|  | Sham (rotation 90°) | MDD | DSM-IV | Yes | 15/14 | 6/9 | 53.3 ± 9.0 | HAMD-21 | 27.4 ± 2.9 | Drug-free | Yes |  |  |  |  |  |  |
| Struckmann et al., 2020, Sweden^[88]^ | iTBS | Mixed | DSM-IV | Yes | 18/18 | 8/10 | 30.0 ± 11.0 | MADRS | 30.0 ± 9.0 | Stable Psychotropic drugs | No | DMPFC | 50 Hz bursts at 5 Hz | 90% | 1,200 (20–30) | 24,000–36,000 | 10–15 days |
|  | Matched sham coil | Mixed | DSM-IV | Yes | 21/21 | 10/11 | 29.0 ± 9.0 | MADRS | 30.0 ± 8.0 | Stable Psychotropic drugs | No |  |  |  |  |  |  |
| Su et al., 2005, China^[89]^ | rTMS | Mixed | DSM-IV | Yes | 10/10 | 3/7 | 43.6 ± 12.0 | HAMD-21 | 23.2 ± 7.5 | Stable Psychotropic drugs | No | Left DLPFC | 20 Hz | 100% | 1,600 (10) | 16,000 | 2 weeks |
|  | rTMS | Mixed | DSM-IV | Yes | 10/10 | 2/8 | 43.2 ± 10.6 | HAMD-21 | 26.5 ± 5.2 | Stable Psychotropic drugs | No | Left DLPFC | 5 Hz | 100% | 1,600 (10) | 16,000 | 2 weeks |
|  | Sham (rotation 90°) | Mixed | DSM-IV | Yes | 10/10 | 3/7 | 42.6 ± 11.0 | HAMD-21 | 22.7 ± 4.7 | Stable Psychotropic drugs | No |  |  |  |  |  |  |
| Tavares et al., 2021, Brazil^[90]^ | TBS | Mixed | DSM-IV | No | 42/28 | 11/31 | 40.8 ± 10.0 | MADRS | 35.4 ± 6.5 | Stable Psychotropic drugs | No | Bilateral DLPFC | 50 Hz bursts at 5 Hz | 90% | 3,600 (21) | 756,000 | 6 weeks |
|  | Matched sham coil | Mixed | DSM-IV | No | 48/36 | 15/33 | 38.0 ± 10.9 | MADRS | 34.5 ± 5.7 | Stable Psychotropic drugs | No |  |  |  |  |  |  |
| Theleritis et al., 2017, Greece^[91]^ | rTMS | MDD | DSM-IV | Yes | 27/25 | 11/16 | 39.1 ± 10.1 | HAMD-17 | 30.6 ± 3.2 | Stable Psychotropic drugs | No | Left DLPFC | 20 Hz | 100% | 1,600 (15) | 24,000 | 3 weeks |
|  | Sham (rotation 90°) | MDD | DSM-IV | Yes | 20/18 | 10/10 | 38.0 ± 9.9 | HAMD-17 | 29.4 ± 3.2 | Stable Psychotropic drugs | No |  |  |  |  |  |  |
|  | rTMS | MDD | DSM-IV | Yes | 27/25 | 15/12 | 38.9 ± 13.9 | HAMD-17 | 29.7 ± 4.6 | Stable Psychotropic drugs | No | Left DLPFC | 20 Hz | 100% | 1,600 (30) | 48,000 | 3 weeks |
|  | Sham (rotation 90°) | MDD | DSM-IV | Yes | 24/21 | 14/10 | 39.4 ± 8.9 | HAMD-17 | 30.3 ± 3.6 | Stable Psychotropic drugs | No |  |  |  |  |  |  |
| Tong et al., 2021, China^[92]^ | rTMS | MDD | DSM-V | No | 60/55 | 24/36 | 48.7 ± 14.0 | HAMD-17 | 29.6 ± 3.6 | Escitalopram | No | Left DLPFC | 10 Hz | 120% | 2,000 (20) | 40,000 | 4 weeks |
|  | Matched sham coil | MDD | DSM-V | No | 60/54 | 29/31 | 47.8 ± 13.4 | HAMD-17 | 28.5 ± 4.0 | Escitalopram | No |  |  |  |  |  |  |
| Triggs et al., 2010, USA^[93]^ | rTMS | MDD | DSM-IV | Yes | 16/16 | 7/9 | 48.5 ± 10.8 | HAMD-24 | 27.2 ± 4.8 | Stable Psychotropic drugs | No | Right DLPFC | 5 Hz | 100% | 2,000 (10) | 20,000 | 2 weeks |
|  | Matched sham coil | MDD | DSM-IV | Yes | 7/7 | 3/4 | 46.6 ± 20.2 | HAMD-24 | 27.3 ± 2.7 | Stable Psychotropic drugs | No |  |  |  |  |  |  |
|  | rTMS | MDD | DSM-IV | Yes | 18/18 | 4/14 | 46.7 ± 15.3 | HAMD-24 | 28.2 ± 6.0 | Stable Psychotropic drugs | No | Left DLPFC | 5 Hz | 100% | 2,000 (10) | 20,000 | 2 weeks |
|  | Matched sham coil | MDD | DSM-IV | Yes | 7/7 | 5/2 | 41.9 ± 14.1 | HAMD-24 | 27.7 ± 3.5 | Stable Psychotropic drugs | No |  |  |  |  |  |  |
| Tsai et al., 2021, China^[94]^ | Prolonged iTBS | MDD | DSM-IV | Yes | 19/19 | 5/14 | 48.7 ± 14.4 | HAMD-17 | 22.5 ± 3.2 | Stable Psychotropic drugs | No | Left DLPFC | 50 Hz bursts at 5 Hz | 80% | 1,800 (10) | 18,000 | 2 weeks |
|  | rTMS | MDD | DSM-IV | Yes | 20/20 | 6/14 | 49.1 ± 14.8 | HAMD-17 | 22.6 ± 3.3 | Stable Psychotropic drugs | No | Left DLPFC | 10 Hz | 100% | 1,600 (10) | 16,000 | 2 weeks |
|  | Matched sham coil | MDD | DSM-IV | Yes | 22/22 | 6/16 | 48.5 ± 12.9 | HAMD-17 | 22.6 ± 2.6 | Stable Psychotropic drugs | No |  |  |  |  |  |  |
| Tura et al., 2024, Italy^[95]^ | Accelerated iTBS | MDD | DSM-V | No | 39/39 | 19/20 | 35.8 ± 13.0 | MADRS | 23.2 ± 7.4 | Stable Antidepressants | No | Left DLPFC | 50 Hz bursts at 5 Hz | 110% | 1,800 (20) | 36,000 | 5 days |
|  | Matched sham coil | MDD | DSM-V | No | 38/38 | 25/13 | 34.9 ± 12.6 | MADRS | 23.9 ± 7.2 | Stable Antidepressants | No |  |  |  |  |  |  |
| Ullrich et al., 2012, Germany^[96]^ | rTMS | MDD | DSM-V | No | 22/22 | 7/15 | 56.9 ± 10.2 | HAMD-21 | 30.4 ± 4.8 | Stable Psychotropic drugs | No | Left DLPFC | 30 Hz | 110% | 1,800 (15) | 27,000 | 3 weeks |
|  | Low-frequency rTMS as sham | MDD | DSM-V | No | 21/21 | 9/12 | 54.1 ± 7.8 | HAMD-21 | 28.2 ± 3.9 | Stable Psychotropic drugs | No | Left DLPFC | 1 Hz | 110% | 1,800 (15) | 27,000 | 3 weeks |
| Wang et al., 2017, China^[97]^ | rTMS | MDD | DSM-IV | No | 22/19 | 12/10 | 28.8 ± 8.4 | HAMD-24 | 43.5 ± 9.9 | Paroxetine | No | Left DLPFC | 10 Hz | 80% | 800 (20) | 16,000 | 4 weeks |
|  | Matched sham coil | MDD | DSM-IV | No | 21/19 | 11/10 | 30.1 ± 9.4 | HAMD-24 | 42.8 ± 9.3 | Paroxetine | No |  |  |  |  |  |  |
| Wang et al., 2021, China^[98]^ | rTMS | MDD | DSM-V | No | 33/32 | 13/19 | 24.9 ± 9.5 | HAMD-17 | 16.1 ± 7.2 | Stable Psychotropic drugs | No | Left DLPFC | 10 Hz | 100% | 3,000 (15) | 45,000 | 15 days |
|  | Matched sham coil | MDD | DSM-V | No | 27/24 | 11/13 | 23.8 ± 8.1 | HAMD-17 | 13.3 ± 5.7 | Stable Psychotropic drugs | No |  |  |  |  |  |  |
| Wilkening et al., 2022, Germany^[99]^ | iTBS | MDD | DSM-IV | No | 40/35 | 21/19 | 35.7 ± 13.0 | MADRS | 23.2 ± 7.6 | Stable Psychotropic drugs | No | Left DLPFC | 50 Hz bursts at 5 Hz | 110% | 1,800 (20) | 36,000 | 5 days |
|  | Matched sham coil | MDD | DSM-IV | No | 41/35 | 26/15 |  | MADRS | 23.2 ± 7.6 | Stable Psychotropic drugs | No |  |  |  |  |  |  |
| Yesavage et al., 2018, USA^[100]^ | rTMS | MDD | DSM-IV | Yes | 81/60 | 67/14 | 55.6 ± 12.2 | HAMD-24 | 26.2 ± 4.9 | Stable Psychotropic drugs | No | Left DLPFC | 10 Hz | 120% | 4,000 (20–30) | 80,000–120,000 | 4–6 weeks |
|  | Matched sham coil | MDD | DSM-IV | Yes | 83/65 | 65/18 | 54.8 ± 12.6 | HAMD-24 | 27.5 ± 5.1 | Stable Psychotropic drugs | No |  |  |  |  |  |  |
| Yildiz et al., 2023, Turkey^[101]^ | rTMS | MDD | DSM-IV | Yes | 15/15 | 2/13 | 40.6 ± 7.2 | HAMD-17 | 22.2 ± 4.9 | Stable Antidepressants | No | Left DLPFC | 10 Hz | 110% | 1,000 (20) | 20,000 | 4 weeks |
|  | Matched sham coil | MDD | DSM-IV | Yes | 15/15 | 6/9 | 37.7 ± 9.3 | HAMD-17 | 20.2 ± 5.0 | Stable Antidepressants | No |  |  |  |  |  |  |
| Yu et al., 2022, China^[102]^ | rTMS | MDD | DSM-IV | No | 25/23 | 9/14 | 23.6 ± 8.4 | HAMD-17 | 14.7 ± 5.3 | Stable Antidepressants | No | Left DLPFC | 10 Hz | 100% | 3,000 (15) | 45,000 | 15 days |
|  | Matched sham coil | MDD | DSM-IV | No | 24/21 | 9/12 | 22.1 ± 4.7 | HAMD-17 | 15.0 ± 5.3 | Stable Antidepressants | No |  |  |  |  |  |  |
| Zavorotnyy et al., 2020, Germany^[103]^ | iTBS | MDD | DSM-IV | No | 28/26 | 14/14 | 40.0 ± 12.1 | HAMD-21 | 16.8 ± 5.9 | Stable Psychotropic drugs | No | Left DLPFC | 50 Hz bursts at 5 Hz | 90% | 1,200 (20) | 24,000 | 2 weeks |
|  | Matched sham coil | MDD | DSM-IV | No | 29/27 | 13/16 | 45.6 ± 12.9 | HAMD-21 | 16.3 ± 4.9 | Stable Psychotropic drugs | No |  |  |  |  |  |  |
| Zengin et al., 2022, Turkey^[104]^ | rTMS | BD | DSM-V | No | 14/14 | 6/8 | 42.4 ± 9.5 | HAMD | 20.4 ± 2.8 | Stable Psychotropic drugs | No | Left DLPFC | 10 Hz | 110% | 1,000 (20) | 20,000 | 4 weeks |
|  | Sham (rotation 45°) | BD | DSM-V | No | 15/15 | 8/7 | 38.9 ± 10.3 | HAMD | 20.1 ± 2.6 | Stable Psychotropic drugs | No |  |  |  |  |  |  |
| Zhang et al., 2021, China^[105]^ | rTMS | MDD | DSM-IV | No | 27/27 | 10/17 | 31.3 ±12.8 | HAMD-24 | 35.8 ± 7.9 | Sleep aid medication | Yes | Left DLPFC | 10 Hz | 90% | 1,600 (10) | 16,000 | 5 days |
|  | Sham (rotation 90°) | MDD | DSM-IV | No | 23/23 | 10/13 | 31.6 ± 10.5 | HAMD-24 | 35.7 ± 9.3 | Sleep aid medication | Yes |  |  |  |  |  |  |
| Zheng et al., 2010, China^[106]^ | rTMS | MDD | DSM-IV | Yes | 19/19 | 12/7 | 26.9 ± 6.2 | HAMD-17 | 24.6 ± 3.0 | Escitalopram | No | Left DLPFC | 15 Hz | 110% | 3,000 (20) | 60,000 | 4 weeks |
|  | Sham (rotation 90°) | MDD | DSM-IV | Yes | 15/15 | 10/5 | 26.7 ± 4.3 | HAMD-17 | 24.6 ± 2.8 | Escitalopram | No |  |  |  |  |  |  |
| Zheng et al., 2015, China^[107]^ | rTMS | MDD | DSM-IV | Yes | 18/18 | 12/6 | 26.9 ± 6.4 | HAMD-17 | 23.1 ± 3.6 | Escitalopram | No | Left DLPFC | 15 Hz | 110% | 3,000 (20) | 20,000 | 4 weeks |
|  | Sham (rotation 90°) | MDD | DSM-IV | Yes | 14/14 | 9/5 | 26.9 ± 4.3 | HAMD-17 | 23.6 ± 3.6 | Escitalopram | No |  |  |  |  |  |  |
| Zhou et al., 2024, China^[108]^ | rTMS | MDD | DSM-IV | No | 60/55 | 26/34 | 32.0 ± 14.5 | HAMD-24 | 26.0 ± 4.5 | Stable Psychotropic drugs | No | Left DLPFC | 10 Hz | 110% | 1,600 (10) | 16,000 | 2 weeks |
|  | Matched sham coil | MDD | DSM-IV | No | 60/53 | 29/31 | 32.7 ± 15.5 | HAMD-24 | 25.9 ± 5.4 | Stable Psychotropic drugs | No |  |  |  |  |  |  |

Note: ^1^ Diagnosis and treatment-resistance status were coded separately and were not mutually exclusive. ^2^ In the present coding framework, H1 deep TMS was classified as left PFC, reflecting predominant left prefrontal stimulation with broader bilateral/medial engagement.

**Table S2**. Quality assessment of the included articles.

| Author, year | Sequence generation | Allocation concealment | Blinding participants and personnel | Blinding of outcome and assessment | Incomplete outcome data | Selective outcome reporting | Overall risk of bias |  |  |  |  |
| --- | --- | --- | --- | --- | --- | --- | --- | --- | --- | --- | --- |
| Akpinar et al., 2022 | Unclear | Unclear | Unclear | Low | Low | Low | Unclear |  |  |  |  |
| Anderson et al., 2007 | Unclear | Low | Unclear | Low | Low | Low | Unclear |  |  |  |  |
| Armas-Castañeda et al., 2021 | Low | Low | Low | Low | Low | Unclear | Low |  |  |  |  |
| Asgharian & Vaghef, 2022 | Unclear | Low | Unclear | Low | Low | Low | Unclear |  |  |  |  |
| Avery et al., 2006 | Low | Unclear | Low | Low | Low | Low | Low |  |  |  |  |
| Bakim et al., 2012 | Low | Unclear | Unclear | Low | Low | Low | Unclear |  |  |  |  |
| Bengtsson et al., 2023 | Low | Low | Low | Low | Low | Low | Low |  |  |  |  |
| Bengtsson et al., 2023 | Low | Low | Low | Low | Low | Low | Low |  |  |  |  |
| Berman et al., 2000 | Unclear | Unclear | Low | Low | High | Low | High |  |  |  |  |
| Blumberger et al., 2012 | Low | Low | Low | Low | Low | Low | Low |  |  |  |  |
| Boutros et al., 2002 | Low | Unclear | Low | Low | Low | Low | Low |  |  |  |  |
| Bretlau et al., 2008 | Unclear | Unclear | Unclear | Unclear | Low | Low | Unclear |  |  |  |  |
| Buchholtz Hansen et al., 2004 | Unclear | Low | Unclear | Low | Low | Low | Unclear |  |  |  |  |
| Carpenter et al., 2017 | Low | Low | Low | Low | Low | Low | Low |  |  |  |  |
| Chen et al., 2013 | Low | Unclear | Low | Low | Unclear | Low | Unclear |  |  |  |  |
| Cheng et al., 2022 | Low | Low | Low | Low | Low | Low | Low |  |  |  |  |
| Cheng et al., 2022 | Low | Low | Low | Low | Low | Low | Low |  |  |  |  |
| Chistyakov et al., 2015 | Unclear | Unclear | Low | Low | Low | Low | Unclear |  |  |  |  |
| Chou et al., 2020 | Unclear | Unclear | Low | Low | Low | Low | Low |  |  |  |  |
| Chou et al., 2023 | Low | Low | Unclear | Low | Low | Low | Low |  |  |  |  |
| Cole et al., 2022 | Low | Low | Low | Low | Low | Low | Low |  |  |  |  |
| Concerto et al., 2015 | Unclear | Unclear | Unclear | Unclear | Low | Low | Unclear |  |  |  |  |
| Dai et al., 2021 | Low | Unclear | Low | Low | Low | Low | Low |  |  |  |  |
| Dellink et al., 2024 | Unclear | Unclear | Low | Low | Low | Low | Low |  |  |  |  |
| Dunlop et al., 2020 | Unclear | Unclear | Low | Low | Low | Low | Low |  |  |  |  |
| Duprat et al., 2016 | Unclear | Unclear | Unclear | Low | Low | Low | Unclear |  |  |  |  |
| Eschweiler et al., 2000 | Unclear | Unclear | Low | Low | Low | Low | Unclear |  |  |  |  |
| Fitzgerald et al., 2003 | Unclear | Low | Low | Low | Low | Low | Unclear |  |  |  |  |
| Fitzgerald et al., 2006 | Low | Low | Low | Low | Low | Low | Low |  |  |  |  |
| Fitzgerald et al., 2012 | Low | Low | Low | Low | Low | Low | Low |  |  |  |  |
| Fitzgerald et al., 2016 | Low | Unclear | Low | Low | Low | Low | Low |  |  |  |  |
| Frick et al., 2021 | Low | Low | Low | Low | Low | Low | Low |  |  |  |  |
| García-Toro et al., 2001 | Unclear | Unclear | Unclear | Low | Unclear | Low | Unclear |  |  |  |  |
| García-Toro et al., 2001 | Unclear | High | Unclear | Low | Unclear | Low | High |  |  |  |  |
| Garcia-Toro et al., 2006 | Unclear | High | Unclear | Low | Low | Low | High |  |  |  |  |
| George et al., 1997 | Unclear | Unclear | Unclear | Low | Low | Unclear | Unclear |  |  |  |  |
| George et al., 2000 | Unclear | Unclear | Low | Low | Low | Low | Unclear |  |  |  |  |
| George et al., 2010 | Low | Unclear | Low | Low | Low | Low | Low |  |  |  |  |
| Hernández-Ribas et al., 2012 | Unclear | Unclear | Unclear | Low | Unclear | Low | Unclear |  |  |  |  |
| Herwig et al., 2003 | Unclear | Unclear | Unclear | Low | Unclear | Low | Unclear |  |  |  |  |
| Herwig et al., 2007 | Low | Unclear | Unclear | Low | Low | Low | Unclear |  |  |  |  |
| Holtzheimer et al., 2004 | Unclear | Unclear | Unclear | Low | Low | Low | Unclear |  |  |  |  |
| Höppner et al., 2003 | Low | Unclear | Unclear | Unclear | Unclear | Low | Unclear |  |  |  |  |
| Hu et al., 2016 | Unclear | Unclear | Unclear | Unclear | Low | Low | Unclear |  |  |  |  |
| Huang et al., 2012 | Low | Unclear | Unclear | Low | Low | Low | Unclear |  |  |  |  |
| Januel et al., 2006 | Low | Unclear | Unclear | Low | High | Low | High |  |  |  |  |
| Kauffmann et al., 2004 | Unclear | Unclear | Unclear | Low | Low | Low | Unclear |  |  |  |  |
| Kazemi et al., 2022 | Low | Unclear | Low | Low | Low | Low | Low |  |  |  |  |
| Koerselman et al., 2004 | Unclear | Unclear | Low | Low | Low | Low | Unclear |  |  |  |  |
| Kreuzer et al., 2015 | Low | Unclear | Low | Low | High | Low | High |  |  |  |  |
| Krstić et al., 2014 | Unclear | Unclear | Unclear | Low | Low | Low | Unclear |  |  |  |  |
| Levkovitz et al., 2015 | Low | Low | Low | Low | Low | Low | Low |  |  |  |  |
| Li et al., 2014 | Unclear | Unclear | Unclear | Low | Low | Low | Unclear |  |  |  |  |
| Li et al., 2021 | Low | Low | Low | Low | Low | Low | Low |  |  |  |  |
| Li et al., 2023 | Low | Low | Low | Low | Low | Low | Low |  |  |  |  |
| Li et al., 2023 | Low | Low | Low | Low | Low | Low | Low |  |  |  |  |
| Li et al., 2024 | Low | Low | Low | Low | Low | Low | Low |  |  |  |  |
| Lingeswaran, 2011 | Low | Unclear | Low | Low | Unclear | Low | Unclear |  |  |  |  |
| Loo et al., 2003 | Unclear | Unclear | Unclear | Low | Low | Low | Unclear |  |  |  |  |
| Loo et al., 2007 | Low | Unclear | Unclear | Low | Low | Low | Unclear |  |  |  |  |
| Mak et al., 2021 | Low | Low | Unclear | Low | Low | Low | Low |  |  |  |  |
| Mallik et al., 2022 | Low | Low | Unclear | Low | Low | Low | Low |  |  |  |  |
| Martiny et al., 2010 | Low | Low | Low | Low | Low | Unclear | Low |  |  |  |  |
| Matsuda et al., 2020 | Low | Low | Low | Low | Low | Low | Low |  |  |  |  |
| McDonald et al., 2006 | Unclear | Unclear | Unclear | Low | Low | Low | Unclear |  |  |  |  |
| McGirr et al., 2021 | Low | Low | Low | Low | Low | Low | Low |  |  |  |  |
| Mogg et al., 2008 | Low | Low | Low | Low | Low | Low | Low |  |  |  |  |
| Murgaš et al., 2023 | Low | Low | Low | Low | Low | Low | Low |  |  |  |  |
| Nahas et al., 2003 | Low | Unclear | Low | Low | Low | Low | Low |  |  |  |  |
| Novák et al., 2024 | Low | Low | Low | Low | Low | Low | Low |  |  |  |  |
| O'Reardon et al., 2007 | Unclear | Unclear | Low | Low | Low | Low | Unclear |  |  |  |  |
| Ortega Rumi et al., 2005 | Unclear | Unclear | Unclear | Low | Unclear | Low | Unclear |  |  |  |  |
| Padberg et al., 2002 | Unclear | Unclear | Unclear | Low | Low | Low | Unclear |  |  |  |  |
| Pallanti et al., 2010 | Low | Low | Unclear | Low | Low | Low | Unclear |  |  |  |  |
| Pan et al., 2020 | Low | High | Low | Low | Low | Low | High |  |  |  |  |
| Pan et al., 2023 | Low | Low | Low | Low | Low | Low | Low |  |  |  |  |
| Plewnia et al., 2014 | Low | Unclear | Unclear | Low | Unclear | Low | Unclear |  |  |  |  |
| Prasser et al., 2014 | Low | Unclear | Low | Low | Low | Low | Low |  |  |  |  |
| Pu et al., 2022 | Unclear | Unclear | Unclear | Low | Unclear | Low | Unclear |  |  |  |  |
| Ramos et al., 2025 | Low | Unclear | Low | Low | Low | Low | Low |  |  |  |  |
| Ray et al., 2011 | Low | Unclear | Low | Low | Low | Low | Low |  |  |  |  |
| Rossini et al., 2005 | Low | Unclear | Low | Low | Low | Low | Low |  |  |  |  |
| Rossini et al., 2005 | Low | Unclear | Unclear | Low | Low | Low | Unclear |  |  |  |  |
| Rothärmel et al., 2021 | Low | Low | Low | Low | Low | Low | Low |  |  |  |  |
| Sheline et al., 2024 | Low | Low | Low | Low | Low | Low | Low |  |  |  |  |
| Speer et al., 2014 | Unclear | Unclear | Unclear | Low | Low | Low | Unclear |  |  |  |  |
| Stern et al., 2007 | Unclear | Unclear | Unclear | Low | Low | Low | Unclear |  |  |  |  |
| Struckmann et al., 2020 | Low | Low | Low | Low | High | Unclear | High |  |  |  |  |
| Su et al., 2005 | Unclear | Unclear | Unclear | Low | Low | Low | Unclear |  |  |  |  |
| Tavares et al., 2021 | Low | Low | Low | Low | High | Low | High |  |  |  |  |
| Theleritis et al., 2017 | Low | Low | Low | Low | Low | Low | Low |  |  |  |  |
| Tong et al., 2021 | Low | Low | Low | Low | Low | Low | Low |  |  |  |  |
| Triggs et al., 2010 | Unclear | Unclear | Low | Low | Low | Low | Unclear |  |  |  |  |
| Tsai et al., 2021 | Unclear | Unclear | Unclear | Low | Low | Low | Unclear |  |  |  |  |
| Tura et al., 2024 | Low | Low | Unclear | Low | Low | Low | Low |  |  |  |  |
| Ullrich et al., 2012 | Unclear | Unclear | Low | Low | Low | Low | Unclear |  |  |  |  |
| Wang et al., 2017 | Low | Low | Low | Low | Low | Low | Low |  |  |  |  |
| Wang et al., 2021 | Low | Unclear | Low | Low | High | Low | High |  |  |  |  |
| Wilkening et al., 2022 | Low | Low | Low | Low | Low | Low | Low |  |  |  |  |
| Yesavage et al., 2018 | Low | Low | Low | Low | Low | Low | Low |  |  |  |  |
| Yildiz et al., 2023 | Low | Low | Low | Low | Low | Low | Low |  |  |  |  |
| Yu et al., 2022 | Unclear | Unclear | Low | Low | Low | Low | Low |  |  |  |  |
| Zavorotnyy et al., 2020 | Unclear | Unclear | Low | Low | Low | Low | Low |  |  |  |  |
| Zengin et al., 2022 | Low | Low | Low | Low | Low | Low | Low |  |  |  |  |
| Zhang et al., 2021 | Low | Low | Low | Low | Low | Low | Low |  |  |  |  |
| Zheng et al., 2010 | Unclear | Unclear | Unclear | Unclear | Unclear | Low | Unclear |  |  |  |  |
| Zheng et al., 2015 | Unclear | Unclear | Unclear | Low | Low | Low | Unclear |  |  |  |  |
| Zhou et al., 2024 | Low | Low | Low | Low | Low | Low | Low |  |  |  |  |

**Table S3**. Dose-response associations of rTMS with total pulses for depressive symptoms, treatment response, and remission.

| Outcome type | Studies (values) | ED_max_ (95% CI) | Max effect (95% CI) | Overall test (χ², df, p) | Non-linearity test (p) | Adjusted R² |
| --- | --- | --- | --- | --- | --- | --- |
| Symptom score |  |  |  |  |  |  |
| Acute | 105 (126) | 30,835 (16,072–45,597) | SMD 0.73 (0.58–0.88) | 90.42, (2), <0.0001 | 0.001 | 0.413 |
| Follow-up | 30 (35) | 26,404 (6,888–45,921) | SMD 1.07 (0.54–1.60) | 16.01, (2), 0.0003 | 0.080 | 0.300 |
| Response |  |  |  |  |  |  |
| Acute | 87 (100) | 37,043 (30,683–43,403) | RR 2.61 (2.18–3.16) | 108.33, (2), <0.0001 | <0.001 | 0.494 |
| Follow-up | 14 (16) | 32,334 (24,995–39,672) | RR 2.12 (1.32–3.35) | 10.10, (2), 0.006 | 0.016 | 0.130 |
| Remission |  |  |  |  |  |  |
| Acute | 58 (72) | 39,053 (31,994–46,111) | RR 2.77 (2.10–3.67) | 51.40, (2), <0.0001 | <0.001 | 0.366 |
| Follow-up | 11 (11) | 50,375 (11,097–91,847) | RR 1.48 (1.06–2.03) | 6.60, (2), 0.037 | 0.277 | 0.109 |

**Table S4**. Dose-response associations of rTMS with pulses per session for depressive symptoms, treatment response, and remission.

| Outcome type | Studies (values) | ED_max_ (95% CI) | Max effect (95% CI) | Overall test (χ², df, p) | Non-linearity test (p) | Adjusted R² |
| --- | --- | --- | --- | --- | --- | --- |
| Symptom score |  |  |  |  |  |  |
| Acute | 105 (126) | 1,807 (873–2,741) | SMD 0.73 (0.59–0.87) | 106.21, (2), <0.0001 | 0.0007 | 0.434 |
| Follow-up | 30 (35) | 1,275 (872–1,678) | SMD 1.23 (0.63–1.83) | 25.03, (2), <0.0001 | 0.031 | 0.376 |
| Response |  |  |  |  |  |  |
| Acute | 87 (100) | 2,169 (1,829–2,509) | RR 2.56 (2.14–3.03) | 109.40, (2), <0.0001 | <0.001 | 0.535 |
| Follow-up | 14 (16) | 1,616 (1,476–1,756) | RR 2.12 (1.43–3.16) | 13.98, (2), 0.0009 | 0.0016 | 0.046 |
| Remission |  |  |  |  |  |  |
| Acute | 58 (72) | 1801 (1,606–1,996) | RR 2.83 (2.27–3.56) | 83.65, (2), <0.0001 | <0.001 | 0.434 |
| Follow-up | 11 (11) | 1,840 (1,488–2,193) | RR 1.48 (1.13–1.92) | 8.02, (2), 0.018 | 0.028 | 0.376 |

**Table S5**. Dose-response associations of rTMS with total sessions for depressive symptoms, treatment response, and remission.

| Outcome type | Studies (values) | ED_max_ (95% CI) | Max effect (95% CI) | Overall test (χ², df, p) | Non-linearity test (p) | Adjusted R² |
| --- | --- | --- | --- | --- | --- | --- |
| Symptom score |  |  |  |  |  |  |
| Acute | 105 (126) | 14 (11–17) | SMD 0.81 (0.64–0.98) | 103.34, (2), <0.0001 | <0.001 | 0.434 |
| Follow-up | 30 (35) | –– | SMD 1.05 (0.57–1.52) | 26.01, (2), <0.0001 | 0.138 | 0.322 |
| Response |  |  |  |  |  |  |
| Acute | 87 (100) | 16 (12–19) | RR 2.29 (1.90–2.77) | 97.14, (2), <0.0001 | 0.0001 | 0.411 |
| Follow-up | 14 (16) | 14 (12–16) | RR 1.97 (1.22–3.16) | 9.87, (2), 0.007 | 0.060 | 0.098 |
| Remission |  |  |  |  |  |  |
| Acute | 58 (72) | 23 (17–29) | RR 2.83 (2.27–3.56) | 53.10, (2), <0.0001 | 0.003 | 0.393 |
| Follow-up | 11 (11) | – (linear, no clear peak) | – (trend not significant) | 5.09, (2), 0.078 | 0.955 | 0.322 |

**Table S6**. Dose-response associations of rTMS with treatment duration (week) for depressive symptoms, treatment response, and remission.

| Outcome type | Studies (values) | ED_max_ (95% CI) | Max effect (95% CI) | Overall test (χ², df, p) | Non-linearity test (p) | Adjusted R² |
| --- | --- | --- | --- | --- | --- | --- |
| Symptom score |  |  |  |  |  |  |
| Acute | 105 (126) | 2.9 (2.4–3.5) | SMD 0.84 (0.67–1.00) | 105.72, (2), <0.0001 | <0.001 | 0.450 |
| Follow-up | 30 (35) | 2.8 (2.5–3.5) | SMD 1.36 (0.83–1.88) | 25.88, (2), <0.0001 | 0.138 | 0.339 |
| Response |  |  |  |  |  |  |
| Acute | 87 (100) | 2.7 (2.2–3.2) | RR 2.39 (1.99–2.89) | 112.46, (2), <0.0001 | <0.001 | 0.478 |
| Follow-up | 14 (16) | 2.9 (2.4–3.5) | RR 2.03 (1.36–3.00) | 12.54, (2), 0.007 | 0.002 | 0.005 |
| Remission |  |  |  |  |  |  |
| Acute | 58 (72) | 3.1 (2.5–3.7) | RR 2.56 (2.01–3.32) | 62.80, (2), <0.0001 | <0.001 | 0.455 |
| Follow-up | 11 (11) | 3.3 (2.6–4.0) | RR 1.36 (1.12–1.66) | 9.33, (2), 0.078 | 0.009 | 0.282 |

**Table S7**. Dose-response associations of rTMS subgroup with total pulses for depressive symptoms, treatment response, and remission.

| Outcome type | Studies (values) | ED_max_ (95% CI) | Max effect (95% CI) | Overall test (χ², df, p) | Non-linearity test (p) | Adjusted R² |
| --- | --- | --- | --- | --- | --- | --- |
| Symptom score |  |  |  |  |  |  |
| Acute | 84 (98) | 28,895 (15,520–42,271) | SMD 0.81 (0.62–0.99) | 74.31 (2), <0.0001 | <0.001 | 0.428 |
| Follow-up | 26 (30) | 24488 (9,147–39,828) | SMD 1.12 (0.50–1.74) | 14.10 (2), 0.0009 | 0.091 | 0.261 |
| Response |  |  |  |  |  |  |
| Acute | 72 (79) | 38,668 (33,019–44,317) | RR 2.53 (2.12–3.06) | 95.70 (2), <0.0001 | <0.001 | 0.545 |
| Follow-up | 10 (11) | 26,501 (12,327–40,674) | RR 1.90 (1.19–3.03) | 9.01 (2), 0.011 | 0.162 | 0.029 |
| Remission |  |  |  |  |  |  |
| Acute | 49 (60) | 38,170 (33,747–42,592) | RR 3.00 (2.20–4.14) | 49.38 (2), <0.0001 | <0.001 | 0.452 |
| Follow-up | 8 (8) | 46,687 (6,059–87,315) | RR 1.39 (1.07–1.80) | 6.06 (2), 0.048 | 0.148 | 0.434 |

**Table S8**. Dose-response associations of TBS subgroup with total pulses for depressive symptoms, treatment response, and remission.

| Outcome type | Studies (values) | ED_max_ (95% CI) | Max effect (95% CI) | Overall test (χ², df, p) | Non-linearity test (p) | Adjusted R² |
| --- | --- | --- | --- | --- | --- | --- |
| Symptom score |  |  |  |  |  |  |
| Acute | 26 (29) | – | – | 32.08 (2), <0.0001 | 0.029 | 0.474 |
| Follow-up | 5 (5) | – | – | 21.08 (2), <0.0001 | 0.368 | 0.860 |
| Response |  |  |  |  |  |  |
| Acute | 20 (21) | 40,996 (26,031–55,960) | RR 2.92 (1.82–4.62) | 22.25 (2), <0.0001 | 0.041 | 0.266 |
| Follow-up | 5 (5) | 40839 (32,466–49,212) | RR 5.05 (1.55–16.28) | 7.32 (2), 0.026 | 0.029 | −0.176 |
| Remission |  |  |  |  |  |  |
| Acute | 12 (12) | 58,813 (-63,518–181,143) | RR 2.27 (1.23–4.22) | 7.64 (2), 0.022 | 0.501 | 0.030 |
| Follow-up | 3 (3) | – | – | 2.84 (2), 0.241 | 0.194 | −0.974 |

**Table S9**. Dose-response associations of rTMS subgroup with pulses per session for depressive symptoms, treatment response, and remission.

| Outcome type | Studies (values) | ED_max_ (95% CI) | Max effect (95% CI) | Overall test (χ², df, p) | Non-linearity test (p) | Adjusted R² |
| --- | --- | --- | --- | --- | --- | --- |
| Symptom score |  |  |  |  |  |  |
| Acute | 84 (98) | 1,564 (1,041–2,087) | SMD 0.72 (0.54–0.89) | 73.44 (2), <0.0001 | 0.002 | 0.432 |
| Follow-up | 26 (30) | 1,254 (922–1,586) | SMD 1.15 (0.46–1.84) | 16.20 (2), 0.0003 | 0.046 | 0.322 |
| Response |  |  |  |  |  |  |
| Acute | 72 (79) | 2,340 (1,729–2,952) | RR 2.43 (2.05–2.89) | 104.52 (2), <0.0001 | <0.001 | 0.570 |
| Follow-up | 10 (11) | – | – | 7.69 (2), 0.021 | 0.296 | −0.117 |
| Remission |  |  |  |  |  |  |
| Acute | 49 (60) | 1,852 (1,628–2,075) | RR 2.94 (2.32–3.74) | 77.71 (2), <0.0001 | <0.001 | 0.506 |
| Follow-up | 8 (8) | 2,245 (-276–4,765) | RR 1.31 (1.05–1.62) | 5.94 (2), 0.051 | 0.321 | 0.419 |

**Table S10**. Dose-response associations of TBS subgroup with pulses per session for depressive symptoms, treatment response, and remission.

| Outcome type | Studies (values) | ED_max_ (95% CI) | Max effect (95% CI) | Overall test (χ², df, p) | Non-linearity test (p) | Adjusted R² |
| --- | --- | --- | --- | --- | --- | --- |
| Symptom score |  |  |  |  |  |  |
| Acute | 26 (29) | – | – | 40.67 (2), <0.0001 | 0.130 | 0.519 |
| Follow-up | 5 (5) | – | – | 20.13 (2), <0.0001 | 0.370 | 0.739 |
| Response |  |  |  |  |  |  |
| Acute | 20 (21) | 2,055 (1,714–2,395) | RR 3.46 (2.29–5.26) | 35.16 (2), <0.0001 | 0.004 | 0.592 |
| Follow-up | 5 (5) | 1,889 (1,817–1,961) | RR 3.49 (2.14–5.75) | 25.68 (2), <0.0001 | <0.001 | 0.981 |
| Remission |  |  |  |  |  |  |
| Acute | 12 (12) | 2,123 (780–3,465) | RR 3.22 (1.55–6.62) | 10.96 (2), 0.004 | 0.238 | 0.331 |
| Follow-up | 3 (3) | 1,932 (1,852–2,013) | RR 5.31 (1.92–14.75) | 11.76 (2), 0.003 | <0.001 | 0.981 |

**Table S11**. Dose-response associations of rTMS subgroup with total sessions for depressive symptoms, treatment response, and remission.

| Outcome type | Studies (values) | ED_max_ (95% CI) | Max effect (95% CI) | Overall test (χ², df, p) | Non-linearity test (p) | Adjusted R² |
| --- | --- | --- | --- | --- | --- | --- |
| Symptom score |  |  |  |  |  |  |
| Acute | 84 (98) | 17 (15–`19) | SMD 0.89 (0.71–1.07) | 98.22 (2), <0.0001 | 0.011 | 0.462 |
| Follow-up | 26 (30) | – | – | 20.75 (2), <0.0001 | 0.201 | 0.304 |
| Response |  |  |  |  |  |  |
| Acute | 72 (79) | 15 (14–`16) | RR 2.36 (1.92–2.94) | 88.49 (2), <0.0001 | <0.001 | 0.476 |
| Follow-up | 10 (11) | – | – | 5.87 (2), 0.053 | 0.447 | −0.006 |
| Remission |  |  |  |  |  |  |
| Acute | 49 (60) | 16 (15–`17) | RR 2.86 (2.18–3.71) | 72.27 (2), <0.0001 | <0.001 | 0.567 |
| Follow-up | 8 (8) | 18 (16–`20) | RR 1.28 (1.03–1.58) | 6.03 (2), 0.049 | 0.586 | 0.431 |

**Table S12**. Dose-response associations of TBS subgroup with total sessions for depressive symptoms, treatment response, and remission.

| Outcome type | Studies (values) | ED_max_ (95% CI) | Max effect (95% CI) | Overall test (χ², df, p) | Non-linearity test (p) | Adjusted R² |
| --- | --- | --- | --- | --- | --- | --- |
| Symptom score |  |  |  |  |  |  |
| Acute | 26 (29) | 10 (7–`13) | SMD 0.61 (0.37–0.86) | 26.54 (2), <0.0001 | 0.0004 | 0.422 |
| Follow-up | 5 (5) | 8 (5–`11) | SMD 1.50 (0.65–2.34) | 19.96 (2), <0.0001 | 0.010 | 0.494 |
| Response |  |  |  |  |  |  |
| Acute | 20 (21) | 15 (12–`18) | RR 3.63 (1.86–7.10) | 19.09 (2), 0.0001 | 0.025 | 0.356 |
| Follow-up | 5 (5) | – | – | 4.37 (2), 0.113 | 0.972 | 0.230 |
| Remission |  |  |  |  |  |  |
| Acute | 12 (12) | – | – | 10.77 (2), 0.005 | 0.417 | 0.352 |
| Follow-up | 3 (3) | – | – | 0.30 (2), 0.862 | 0.827 | −0.270 |

**Table S13**. Dose-response associations of rTMS subgroup with treatment duration (week) for depressive symptoms, treatment response, and remission.

| Outcome type | Studies (values) | ED_max_ (95% CI) | Max effect (95% CI) | Overall test (χ², df, p) | Non-linearity test (p) | Adjusted R² |
| --- | --- | --- | --- | --- | --- | --- |
| Symptom score |  |  |  |  |  |  |
| Acute | 84 (98) | 3.0 (2.5–`3.5) | SMD 0.92 (0.72–1.11) | 86.34 (2), <0.0001 | <0.001 | 0.438 |
| Follow-up | 26 (30) | 2.9 (2.5–`3.3) | SMD 1.28 (0.73–1.84) | 20.83 (2), <0.0001 | 0.163 | 0.310 |
| Response |  |  |  |  |  |  |
| Acute | 72 (79) | 3.0 (2.6–`3.4) | RR 2.23 (1.82–2.69) | 87.22 (2), <0.0001 | <0.001 | 0.481 |
| Follow-up | 10 (11) | – | – | 5.86 (2), 0.053 | 0.447 | −0.006 |
| Remission |  |  |  |  |  |  |
| Acute | 49 (60) | 3 (2.5–`3.5) | RR 2.72 (2.08–3.56) | 64.45 (2), <0.0001 | 0.003 | 0.536 |
| Follow-up | 8 (8) | 3.5 (1.2–`5.8) | RR 1.27 (1.05–1.55) | 6.03 (2), 0.049 | 0.586 | 0.431 |

**Table S14**. Dose-response associations of TBS subgroup with treatment duration (week) for depressive symptoms, treatment response, and remission.

| Outcome type | Studies (values) | ED_max_ (95% CI) | Max effect (95% CI) | Overall test (χ², df, p) | Non-linearity test (p) | Adjusted R² |
| --- | --- | --- | --- | --- | --- | --- |
| Symptom score |  |  |  |  |  |  |
| Acute | 26 (29) | 2.0 (1.8–`2.2) | SMD 0.68 (0.52–0.85) | 68.51 (2), <0.0001 | <0.001 | 0.580 |
| Follow-up | 5 (5) | 2.0 (1.7–`2.3) | SMD 1.45 (0.13–2.77) | 18.92 (2), 0.0001 | 0.349 | 0.596 |
| Response |  |  |  |  |  |  |
| Acute | 20 (21) | 2.0 (1.8–`2.2) | RR 2.92 (1.90–4.48) | 25.01 (2), <0.0001 | <0.001 | 0.509 |
| Follow-up | 5 (5) | – | – | 20.89 (2), <0.0001 | 0.359 | 0.786 |
| Remission |  |  |  |  |  |  |
| Acute | 12 (12) | 2.5 (1.5–`3.5) | RR 2.69 (1.26–5.81) | 6.47 (2), 0.039 | 0.036 | 0.124 |
| Follow-up | 3 (3) | – | – | 1.89 (2), 0.389 | 0.198 | 0.059 |

**Table S15**. Dose-response associations of rTMS with total pulses in unipolar depression subgroup for depressive symptoms, treatment response, and remission.

| Outcome type | Studies (values) | ED_max_ (95% CI) | Max effect (95% CI) | Overall test (χ², df, p) | Non-linearity test (p) | Adjusted R² |
| --- | --- | --- | --- | --- | --- | --- |
| Symptom score |  |  |  |  |  |  |
| Acute | 82 (101) | 41,582 (22,077–61,087) | SMD 1.03 (0.79–1.27) | 70.35, (2), <0.0001 | <0.001 | 0.421 |
| Follow-up | 29 (34) | 25,626 (8,707–42,545) | SMD 1.09 (0.55–1.64) | 15.90, (2), 0.0004 | 0.071 | 0.302 |
| Response |  |  |  |  |  |  |
| Acute | 64 (77) | 43,074 (37,305–48,842) | RR 2.99 (2.42–3.69) | 106.20, (2), <0.0001 | <0.001 | 0.589 |
| Follow-up | 12 (14) | 31,004 (18,264–43,745) | RR 2.17 (1.39–3.39) | 11.68, (2), 0.0029 | 0.050 | 0.164 |
| Remission |  |  |  |  |  |  |
| Acute | 43 (55) | 46,285 (39,297–53,274) | RR 3.22 (2.24–4.62) | 42.36, (2), <0.0001 | <0.001 | 0.393 |
| Follow-up | 9 (9) | – | – | 7.91, (2), 0.0192 | 0.576 | 0.274 |

**Table S16** Dose-response associations of rTMS with pulses per session in unipolar depression subgroup for depressive symptoms, treatment response, and remission.

| Outcome type | Studies (values) | ED_max_ (95% CI) | Max effect (95% CI) | Overall test (χ², df, p) | Non-linearity test (p) | Adjusted R² |
| --- | --- | --- | --- | --- | --- | --- |
| Symptom score |  |  |  |  |  |  |
| Acute | 82 (101) | 1,903 (907–2,898) | SMD 2.08 (1.79–2.41) | 93.22, (2), <0.0001 | 0.010 | 0.432 |
| Follow-up | 29 (34) | 1,258 (930–1,586) | SMD 1.27 (0.65–1.88) | 25.03, (2), <0.0001 | 0.025 | 0.380 |
| Response |  |  |  |  |  |  |
| Acute | 64 (77) | 2,331 (1,837–2,825) | RR 1.56 (0.56–4.33) | 120.10, (2), <0.0001 | <0.001 | 0.615 |
| Follow-up | 12 (14) | 1,650 (1,070–2,230) | RR 2.13 (1.45–3.11) | 16.70, (2), 0.0002 | 0.142 | –0.019 |
| Remission |  |  |  |  |  |  |
| Acute | 43 (55) | 1,928 (1,647–2,208) | RR 2.89 (2.21–3.79) | 60.87, (2), <0.0001 | <0.001 | 0.476 |
| Follow-up | 9 (9) | 2,036 (1,223–2,849) | RR 1.49 (1.14–1.94) | 8.71, (2), 0.0129 | 0.115 | 0.280 |

**Table S17** Dose-response associations of rTMS with total sessions in unipolar depression subgroup for depressive symptoms, treatment response, and remission.

| Outcome type | Studies (values) | ED_max_ (95% CI) | Max effect (95% CI) | Overall test (χ², df, p) | Non-linearity test (p) | Adjusted R² |
| --- | --- | --- | --- | --- | --- | --- |
| Symptom score |  |  |  |  |  |  |
| Acute | 82 (101) | 14 (11–17) | SMD 0.91 (0.71–1.11) | 88.67, (2), <0.0001 | <0.001 | 0.453 |
| Follow-up | 29 (34) | – | – | 26.83, (2), <0.0001 | 0.124 | 0.328 |
| Response |  |  |  |  |  |  |
| Acute | 64 (77) | 21 (16–26) | RR 2.16 (1.22–3.83) | 95.21, (2), <0.0001 | <0.001 | 0.454 |
| Follow-up | 12 (14) | 14 (11–18) | RR 1.88 (1.21–2.94) | 9.83, (2), 0.0074 | 0.205 | 0.131 |
| Remission |  |  |  |  |  |  |
| Acute | 43 (55) | 22(17–27) | RR 2.82 (2.08–3.83) | 43.74, (2), <0.0001 | 0.001 | 0.437 |
| Follow-up | 9 (9) | – | – | 12.74, (2), 0.0017 | 0.207 | 0.464 |

**Table S18** Dose-response associations of rTMS with total treatment duration (week) in unipolar depression subgroup for depressive symptoms, treatment response, and remission.

| Outcome type | Studies (values) | ED_max_ (95% CI) | Max effect (95% CI) | Overall test (χ², df, p) | Non-linearity test (p) | Adjusted R² |
| --- | --- | --- | --- | --- | --- | --- |
| Symptom score |  |  |  |  |  |  |
| Acute | 82 (101) | 2.8 (2.6–3.1) | SMD 0.92 (0.72–1.13) | 89.83, (2), <0.0001 | <0.001 | 0.456 |
| Follow-up | 29 (34) | 2.8 (2.6–3.1) | SMD 1.41 (0.86–1.94) | 25.55, (2), <0.0001 | 0.022 | 0.352 |
| Response |  |  |  |  |  |  |
| Acute | 64 (77) | 2.7 (2.5–2.9) | RR 2.47 (2.05–2.97) | 101.45, (2), <0.0001 | <0.001 | 0.503 |
| Follow-up | 12 (14) | 2.8 (2.6–3.3) | RR 2.59 (1.46–4.61) | 14.96, (2), 0.0006 | 0.079 | 0.034 |
| Remission |  |  |  |  |  |  |
| Acute | 43 (55) | 3.2 (2.8–3.5) | RR 2.62 (1.98–3.48) | 49.81, (2), <0.0001 | 0.005 | 0.473 |
| Follow-up | 9 (9) | 3.3 (2.3–4.3) | RR 1.35 (1.09–1.69) | 8.76, (2), 0.0125 | 0.329 | 0.230 |

**Table S19**. Tests of Heterogeneity and Publication Bias Across rTMS Dosing Parameters and Clinical Outcomes.

| Stratum | tau^2^ | I^2^_percent | Q_p | Egger_SE_beta | Egger_SE_p |
| --- | --- | --- | --- | --- | --- |
| Total pulses |  |  |  |  |  |
| Acute depressive-symptom severity | 0.581296 | 3.05 | 0.122 | -0.0949 | 0.441 |
| Follow-up depressive-symptom severity | 17.56435 | 59.01 | 1.19E-17 | 0.0997 | 0.774 |
| Acute response | 0.155672 | 50.13 | 3.09E-06 | 0.0465 | 0.856 |
| Follow-up response | 0.201376 | 81.14 | 2.02E-05 | -0.27 | 0.247 |
| Acute remission | 0.041141 | 9.14 | 0.146 | 0.0853 | 0.776 |
| Follow-up remission | 0.119306 | 62.13 | 0.129 | 1.72 | 0.0113 |
| Pulses per session |  |  |  |  |  |
| Acute depressive-symptom severity | 0.581296 | 3.05 | 0.122 | -0.101 | 0.408 |
| Follow-up depressive-symptom severity | 24.0599 | 73.5 | 4.61E-10 | 0.408 | 0.568 |
| Acute response | 0.157629 | 48.45 | 4.10E-06 | 0.408 | 0.568 |
| Follow-up response | 0.201376 | 81.14 | 2.02E-05 | -0.611 | 0.277 |
| Acute remission | 0.041141 | 9.14 | 0.146 | 0.392 | 0.462 |
| Follow-up remission | 0.119306 | 62.13 | 0.129 | 1.74 | 0.00583 |
| Total sessions |  |  |  |  |  |
| Acute depressive-symptom severity | 0.581296 | 3.05 | 0.122 | -0.207 | 0.0953 |
| Follow-up depressive-symptom severity | 17.56435 | 59.01 | 1.19E-17 | 0.0586 | 0.861 |
| Acute response | 0.157629 | 48.45 | 4.10E-06 | -0.197 | 0.461 |
| Follow-up response | 0.201376 | 81.14 | 2.02E-05 | 2.02 | 1.76E-06 |
| Acute remission | 0.041141 | 9.14 | 0.146 | 0.112 | 0.862 |
| Follow-up remission | 0.119306 | 62.13 | 0.129 | 1.31 | 0.0683 |
| Treatment duration |  |  |  |  |  |
| Acute depressive-symptom severity | 0.581296 | 3.05 | 0.122 | -0.209 | 0.0912 |
| Follow-up depressive-symptom severity | 17.56435 | 59.01 | 1.19E-17 | 0.0141 | 0.965 |
| Acute response | 0.157629 | 48.45 | 4.10E-06 | -0.24 | 0.275 |
| Follow-up response | -0.24 | -0.24 | -0.24 | -0.24 | 0.275 |
| Acute remission | 0.041141 | 9.14 | 0.146 | 0.248 | 0.316 |
| Follow-up remission | 0.119306 | 62.13 | 0.129 | 0.789 | 0.100 |

**Fig. S1**. Overview of study characteristics and stimulation parameter distributions among 108 included randomized sham-controlled trials.

A show the flow of TMS treatment modalities and stimulation sites across different types of depressive episodes. Panels B–E summarize stimulation intensity, frequency, treatment duration, and total number of sessions. Panels F–G display the distributions of pulses per session and total pulses across all included trials. BD, bipolar disorder; MDD, major depressive disorder; TRD, treatment-resistant depression; TBS, theta-burst stimulation; rTMS, repetitive transcranial magnetic stimulation; R.PFC, right prefrontal cortex; L.PFC, left prefrontal cortex; R.DLPFC, right dorsolateral prefrontal cortex; L.DLPFC, left dorsolateral prefrontal cortex; DMPFC, dorsomedial prefrontal cortex.

**Fig. S2**. Summary of risk of bias across the 108 included studies.

**Fig. S3**. Dose-response associations of rTMS subgroup with total pulses across depressive-symptom, treatment-response, and remission outcomes.

Panels A–B display standardized mean differences (SMDs) for continuous depressive-symptom scores, and Panels C–F display risk ratios (RR) for binary endpoints (treatment response and remission), during acute and follow-up periods. Curves were fitted using one-stage random-effects restricted cubic spline models with REML estimation. Shaded areas indicate pointwise 95% confidence intervals.

**Fig. S4**. Dose-response associations of rTMS subgroup with pulses per session across depressive-symptom, treatment-response, and remission outcomes.

Panels A–B display standardized mean differences (SMDs) for continuous depressive-symptom scores, and Panels C–F display risk ratios (RR) for binary endpoints (treatment response and remission), during acute and follow-up periods. Curves were fitted using one-stage random-effects restricted cubic spline models with REML estimation. Shaded areas indicate pointwise 95% confidence intervals.

**Fig. S5**. Dose-response associations of rTMS subgroup with total sessions across depressive-symptom, treatment-response, and remission outcomes.

Panels A–B display standardized mean differences (SMDs) for continuous depressive-symptom scores, and Panels C–F display risk ratios (RR) for binary endpoints (treatment response and remission), during acute and follow-up periods. Curves were fitted using one-stage random-effects restricted cubic spline models with REML estimation. Shaded areas indicate pointwise 95% confidence intervals.

**Fig. S6**. Dose-response associations of rTMS subgroup with treatment duration (week) across depressive-symptom, treatment-response, and remission outcomes.

Panels A–B display standardized mean differences (SMDs) for continuous depressive-symptom scores, and Panels C–F display risk ratios (RR) for binary endpoints (treatment response and remission), during acute and follow-up periods. Curves were fitted using one-stage random-effects restricted cubic spline models with REML estimation. Shaded areas indicate pointwise 95% confidence intervals.

**Fig. S7**. Dose-response associations of TBS subgroup with total pulses across depressive-symptom, treatment-response, and remission outcomes.

Panels A–B display standardized mean differences (SMDs) for continuous depressive-symptom scores, and Panels C–F display risk ratios (RR) for binary endpoints (treatment response and remission), during acute and follow-up periods. Curves were fitted using one-stage random-effects restricted cubic spline models with REML estimation. Shaded areas indicate pointwise 95% confidence intervals.

**Fig. S8**. Dose-response associations of TBS subgroup with pulses per session across depressive-symptom, treatment-response, and remission outcomes.

Panels A–B display standardized mean differences (SMDs) for continuous depressive-symptom scores, and Panels C–F display risk ratios (RR) for binary endpoints (treatment response and remission), during acute and follow-up periods. Curves were fitted using one-stage random-effects restricted cubic spline models with REML estimation. Shaded areas indicate pointwise 95% confidence intervals.

V

**Fig. S9**. Dose-response associations of TBS subgroup with total sessions across depressive-symptom, treatment-response, and remission outcomes.

Panels A–B display standardized mean differences (SMDs) for continuous depressive-symptom scores, and Panels C–F display risk ratios (RR) for binary endpoints (treatment response and remission), during acute and follow-up periods. Curves were fitted using one-stage random-effects restricted cubic spline models with REML estimation. Shaded areas indicate pointwise 95% confidence intervals.

**Fig. S10**. Dose-response associations of TBS subgroup with treatment duration (week) across depressive-symptom, treatment-response, and remission outcomes.

Panels A–B display standardized mean differences (SMDs) for continuous depressive-symptom scores, and Panels C–F display risk ratios (RR) for binary endpoints (treatment response and remission), during acute and follow-up periods. Curves were fitted using one-stage random-effects restricted cubic spline models with REML estimation. Shaded areas indicate pointwise 95% confidence intervals.

**Fig. S11**. Dose-response associations of unipolar depression subgroup with total pulses across depressive-symptom, treatment-response, and remission outcomes.

Panels A–B display standardized mean differences (SMDs) for continuous depressive-symptom scores, and Panels C–F display risk ratios (RR) for binary endpoints (treatment response and remission), during acute and follow-up periods. Curves were fitted using one-stage random-effects restricted cubic spline models with REML estimation. Shaded areas indicate pointwise 95% confidence intervals.

**Fig. S12**. Dose-response associations of unipolar depression subgroup with pulses per session across depressive-symptom, treatment-response, and remission outcomes.

Panels A–B display standardized mean differences (SMDs) for continuous depressive-symptom scores, and Panels C–F display risk ratios (RR) for binary endpoints (treatment response and remission), during acute and follow-up periods. Curves were fitted using one-stage random-effects restricted cubic spline models with REML estimation. Shaded areas indicate pointwise 95% confidence intervals.

**Fig. S13**. Dose-response associations of unipolar depression subgroup with total sessions across depressive-symptom, treatment-response, and remission outcomes.

Panels A–B display standardized mean differences (SMDs) for continuous depressive-symptom scores, and Panels C–F display risk ratios (RR) for binary endpoints (treatment response and remission), during acute and follow-up periods. Curves were fitted using one-stage random-effects restricted cubic spline models with REML estimation. Shaded areas indicate pointwise 95% confidence intervals.

**Fig. S14**. Dose-response associations of unipolar depression subgroup with treatment duration (week) across depressive-symptom, treatment-response, and remission outcomes.

Panels A–B display standardized mean differences (SMDs) for continuous depressive-symptom scores, and Panels C–F display risk ratios (RR) for binary endpoints (treatment response and remission), during acute and follow-up periods. Curves were fitted using one-stage random-effects restricted cubic spline models with REML estimation. Shaded areas indicate pointwise 95% confidence intervals.

**Fig. S15**. Leave-one-out sensitivity analyses for total-pulse-based dose-response models of rTMS.

Panels A–E show leave-one-out (LOO) refits of restricted cubic spline models for acute and follow up outcomes (symptom severity, response, and remission), while panels B and F summarize LOO slopes from one-stage random-effects meta-regression models.

**Fig. S16.** Leave-one-out sensitivity analyses for pulses-per-session–based dose–response models of rTMS.

Panels A–F show leave-one-out (LOO) refits of restricted cubic spline models for acute and follow up outcomes (symptom severity, response, and remission).

**Fig. S17**. Leave-one-out sensitivity analyses for total-sessions–based dose–response models of rTMS.

Panels A–E show leave-one-out (LOO) refits of restricted cubic spline models for acute and follow up outcomes (symptom severity, response, and remission), while panels B summarize LOO slopes from one-stage random-effects meta-regression models. **Note:** the follow-up remission model was not significant; therefore, no sensitivity analysis panel is shown for that outcome.

**Fig. S18**. Leave-one-out sensitivity analyses for treatment-duration–based dose–response models of rTMS.

Panels A–F show leave-one-out (LOO) refits of restricted cubic spline models for acute and follow up outcomes (symptom severity, response, and remission), while panels B summarize LOO slopes from one-stage random-effects meta-regression models.

**Fig. S19**. Funnel plots of publication bias for total-pulse–based rTMS dose-response analyses across outcomes.

Panels A–F show results for (A) acute depressive symptom severity, (B) follow-up depressive symptom severity, (C) acute response, (D) follow-up response, (E) acute remission, and (F) follow-up remission.

**Fig. S20**. Funnel plots of publication bias for pulse-per-session–based rTMS dose-response analyses across outcomes.

Panels A–F show results for (A) acute depressive symptom severity, (B) follow-up depressive symptom severity, (C) acute response, (D) follow-up response, (E) acute remission, and (F) follow-up remission.

**Fig. S21**. Funnel plots of publication bias for total-sessions–based rTMS dose-response analyses across outcomes.

Panels A–F show results for (A) acute depressive symptom severity, (B) follow-up depressive symptom severity, (C) acute response, (D) follow-up response, (E) acute remission, and (F) follow-up remission.

**Fig. S22**. Funnel plots of publication bias for treatment-duration–based rTMS dose-response analyses across outcomes.

Panels A–F show results for (A) acute depressive symptom severity, (B) follow-up depressive symptom severity, (C) acute response, (D) follow-up response, (E) acute remission, and (F) follow-up remission.
