# Supplementary material for: Acute and long-term effects of repetitive transcranial magnetic stimulation in major depressive episodes: a systematic review and dose-response meta-analysis of randomized sham-controlled trials
Source: BMC Med. 2026 Jul 30;24:622. doi: 10.1186/s12916-026-05089-z (PMC13746655; doi:10.1186/s12916-026-05089-z)
Supplement: Supplementary file 2 [file 12916_2026_5089_MOESM2_ESM.doc]

| **aSection/topic** | **#** | **Checklist item** | **Reported on page #** |
| --- | --- | --- | --- |
| **TITLE** | | |  |
| Title | 1 | The report is identified as a meta-analysis. | Article Fiile page 1 |
| **ABSTRACT** | | |  |
| Structured summary | 2 | The structured summary includes background, objectives, data sources, study eligibility criteria, participants, and interventions, results, conclusions and implications of key findings. | Article Fiile page 3-4 |
| **INTRODUCTION** | | |  |
| Rationale | 3 | Describe in the introduction. | Article Fiile page 5 |
| Objectives | 4 | Describe in the introduction. | Article Fiile page 5 |
| **METHODS** | | |  |
| Protocol and registration | 5 | The protocol is described in the methods. This study is registered with PROSPERO. | Article Fiile page 6 |
| Eligibility criteria | 6 | Describe in the methods. | Article Fiile page 6-7 |
| Information sources | 7 | Describe in the methods. | Article Fiile page 6 |
| Search | 8 | Describe in the supplementary material eMethods. | Supplementary material eMethods |
| Study selection | 9 | Describe in the methods. | Article Fiile page 6-7 |
| Data collection process | 10 | Describe in the methods. | Article Fiile page 7-8 |
| Data items | 11 | Describe in the supplementary material eMethods. | Supplementary material eMethods |
| Risk of bias in individual studies | 12 | Describe in the methods. | Article Fiile page 9 |
| Summary measures | 13 | Describe in the methods. | Article Fiile page 8-9 |
| Synthesis of results | 14 | The handling data and combining results of studies is described in the methods. The measures of consistency does not apply. | Article Fiile page 8-9 |

Page 1 of 2

| **Section/topic** | **#** | **Checklist item** | **Reported on page #** |
| --- | --- | --- | --- |
| Risk of bias across studies | 15 | The assessment of risk of bias are described in the methods. | Article Fiile page 8 |
| Additional analyses | 16 | The statistical processing of sensitivity are described in the methods. | Article Fiile page 9 |
| **RESULTS** | | |  |
| Study selection | 17 | Describe in the Figure 1. | Figure 1A |
| Study characteristics | 18 | Describe in the Table S1 and Figure 1. | Supplementary material Table S1 and Figure 1 |
| Risk of bias within studies | 19 | The assessment of risk of bias are described in the Figure S1 and Table S2. | Supplementary material Figure S1 and Table S2 |
| Results of individual studies | 20 | Describe in the Table S1 | Supplementary material Table S1 |
| Synthesis of results | 21 | The results of each meta-analysis is descrbied in the results. The confidence intervals and measures of consistency do not apply. | Figures 2-4 |
| Risk of bias across studies | 22 | Describe in the results and supplementary material Table S3-6. | Supplementary material Table S3-6 |
| Additional analysis | 23 | The results of sensitivity analyses and publication bias were described in the results Table S15 and Figure S11-18 | Article Fiile page 13-14, Table S15 and Supplementary material Figures S10-17 |
| **DISCUSSION** | | |  |
| Summary of evidence | 24 | Describe in the discussion. | Article Fiile page 14 |
| Limitations | 25 | Describe in the limitations. | Manuscript page 16-17 |
| Conclusions | 26 | Describe in the conclusions. | Manuscript page 17 |
| **FUNDING** | | |  |
| Funding | 27 | Describe in the funding. | Article Fiile page 1 |

*From:*  Moher D, Liberati A, Tetzlaff J, Altman DG, The PRISMA Group (2009). Preferred Reporting Items for Systematic Reviews and Meta-Analyses: The PRISMA Statement. PLoS Med 6(7): e1000097. doi:10.1371/journal.pmed1000097

For more information, visit: **www.prisma-statement.org**.

Page 2 of 2
